# Transcriptome and Metabolite analysis reveal candidate genes of the cardiac glycoside biosynthetic pathway from *Calotropis procera*

**DOI:** 10.1038/srep34464

**Published:** 2016-10-05

**Authors:** Akansha Pandey, Vishakha Swarnkar, Tushar Pandey, Piush Srivastava, Sanjeev Kanojiya, Dipak Kumar Mishra, Vineeta Tripathi

**Affiliations:** 1 Botany division, CSIR-CDRI, Sector 10, Jankipuram Extension, Sitapur Road, Lucknow 226031, Uttar Pradesh, India; 2 Sophisticated Analytical Instrument Facility, CSIR-CDRI, Sector 10, Jankipuram Extension, Sitapur Road, Lucknow 226031, Uttar Pradesh, India

## Abstract

*Calotropis procera* is a medicinal plant of immense importance due to its pharmaceutical active components, especially cardiac glycosides (CG). As genomic resources for this plant are limited, the genes involved in CG biosynthetic pathway remain largely unknown till date. Our study on stage and tissue specific metabolite accumulation showed that CG’s were maximally accumulated in stems of 3 month old seedlings. *De novo* transcriptome sequencing of same was done using high throughput Illumina HiSeq platform generating 44074 unigenes with average mean length of 1785 base pair. Around 66.6% of unigenes were annotated by using various public databases and 5324 unigenes showed significant match in the KEGG database involved in 133 different pathways of plant metabolism. Further KEGG analysis resulted in identification of 336 unigenes involved in cardenolide biosynthesis. Tissue specific expression analysis of 30 putative transcripts involved in terpenoid, steroid and cardenolide pathways showed a positive correlation between metabolite and transcript accumulation. Wound stress elevated CG levels as well the levels of the putative transcripts involved in its biosynthetic pathways. This result further validated the involvement of identified transcripts in CGs biosynthesis. The identified transcripts will lay a substantial foundation for further research on metabolic engineering and regulation of cardiac glycosides biosynthesis pathway genes.

*Calotropis procera* (Aiton) Dryand is a wild, flowering perennial shrub of family Apocynaceae^1^. It is indigenous to Africa and Asia and naturalized in Australia, Central and Southern America, and the Caribbean island^2^. In traditional Indian medicinal system, *Calotropis* is known to cure diseases like leprosy^3^, ulcers^4^, piles^5^, malaria^6^, snake bite^7^ and several other ailments of spleen and liver^8^. Aqueous extract of flower has analgesic^9^, antipyretic^10^, anti-inflammatory activity^11^, spermicidal activities^12^,^13^ and anti-fungal properties^14^. Recently, these have been rediscovered for their anti-cancer activities in different cancer types^15^. Aqueous extract of *Calotropis procera* latex showed protective effects against hepatocarcinogenesis without any harmful effects in treated animals^16^. Van Quaquebeke reported that a hemi-synthetic derivative (2-Oxovorucharin) isolated from root barks of *C. procera* had a strong *in vitro* cytotoxic effect on several human cancer cell lines^17^. Likewise root extracts of *C. procera* has been shown to inhibit proliferation of Hep-G2 cells through apoptosis and cell cycle disruption based mechanism^18^. Both *in vitro* and *in vivo* growth inhibition of human cancer cell lines by latex proteins from *Calotropis procera* have been studied by Oliveria group^19^. *C. procera* biological activities are attributed to a diverse class of compounds predominately present in form of cardiac glycosides^20^,^21^,^22^, terpenes^23^, flavonoids^24^ and tannin^25^ etc. Many cardiac glycosides have been isolated from *Calotropis procera* like Calactin, Ascelpin, Calotropin, Uscharin, Calotoxin, Uscharidin, Frugoside, Voruscharin^26^,^27^,^28^,^29^. These are known to specifically inhibit the plasma membrane Na^+^/K^+^ ATPase^30^ and have been used in congestive heart failure diseases for decades^31^,^32^.

Cardiac glycosides are characterized by the presence of core steroidal rings with its rings connected cis-trans-cis, possessing a 14β-hydroxyl group, and substituted at C-17β with a lactone ring that constitute aglycone or genin part of cardiac glycosides. At position 3β, a sugar side chain with up to five carbohydrates units is attached which forms glycone part^33^,^34^. The functional groups and sugars attached to the genin unit impart structural diversity of these compounds^35^. These compounds are present in different plant groups and have been well characterized for its chemical structures, but biosynthesis of these compounds have not been studied well till date^36^,^37^. A putative but partial pathway has been derived from precursor feeding studies in *Digitalis lantana* indicating that steroidal framework of cardiac glycosides is derivative of terpenoids, which is supposed to be formed from either mevalonic acid or 2-C-methyl-D-erythritol 4-phosphate. Incorporation of ^14^C-mevalonic acid into steroid part of Digitoxin suggests that it might be the preferred route for formation of genin unit^38^. But contradictory studies do exist postulating that pregnane condensation with acetyl CoA or malonyl CoA, yields cardenolide genin unit^39^. It is suggested that several phytosterols like cholesterol, sitosterol, and stigmasterol can also be metabolized to Pregnenolone, which are thought to be first dedicated step towards cardiac glycosides synthesis. However, evidences regarding involvement of one particular phytosterol (either cholesterol, sitosterol, stigmasterol) and related steroid-backbone biosynthetic genes are still a puzzle to solve^40^,^41^.

Once pregeneolone is formed, it is reduced to Progesterone by action of Hydroxy steroid dehydrogenase (HSD) enzyme^42^. Reduction of Progesterone to Pregnanolone is then catalyzed by Progesterone beta reductase (PBR)^43^. Since cardiac glycosides structures are composed of several hydroxyl groups, it is thought to be the result of direct hydroxylation reaction catalyzed by different Monoxygenases. Enzymes involved in the Pregnane hydroxylation during formation of cardiac glycosides have not yet been identified/characterised^44^,^45^. Apart from this linear pathway described above, Maier *et al.* described an alternative “norcholanic acid pathway” for cardiac glycoside biosynthesis in *Digitalis*^46^. Kreis *et al.* suggested norcholanic acid pathway as precursor funnelling system at final stages of cardenolide biosynthesis^47^. 5β-POR and 3β-HSD which have a well-established role in pregnane pathway were also able to catalyse norcholanic acid and their intermediates^48^,^49^.

As cardiac glycosides compounds have limited natural abundance and there is no successful scheme for their total chemical synthesis^50^, an alternative method for CGs biosynthesis and enrichment is required to meet the raising global demand. *Calotropis procera* have more structurally diverse cardiac glycosides when compared to *Digitalis*, so it’s important to elucidate the biosynthetic pathway from *C. procera* to gain better insight of the process. For the first time, we have studied the accumulation of different CGs in stage and tissue specific manner and identified the putative genes involved in its biosynthesis. Based on the present information and taking leads from the data obtained from KEGG analysis we postulated a putative CG biosynthesis pathway in *Calotropis*. Information regarding the genes or enzymes involved in the biosynthesis of CGs will be very useful for establishing alternate strategies for its increased production.

## Results

### Cardiac Glycoside profiling at different developmental stages

**Figure 1:**
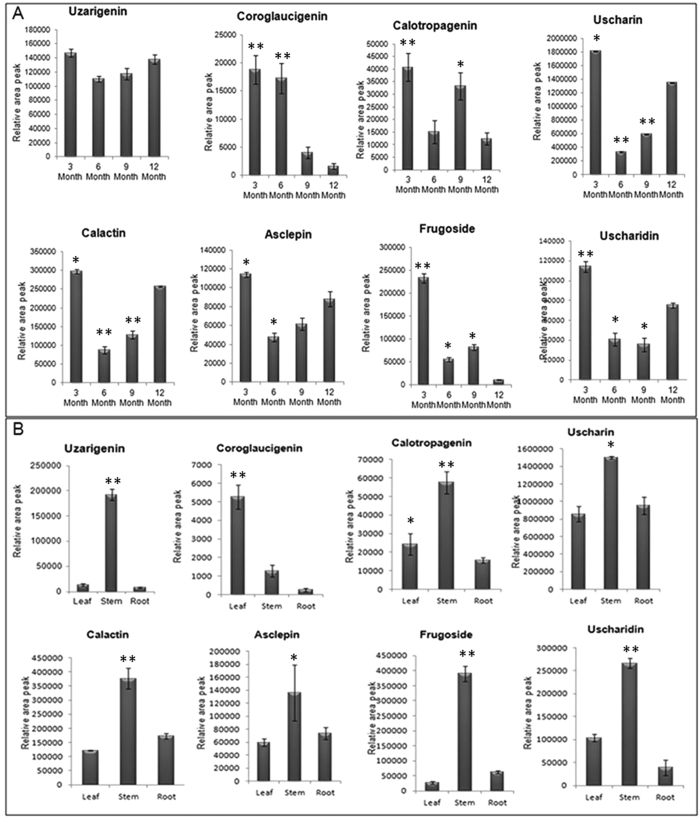
(**A**) Accumulation of CGs and genin units at different stage of *C. procera* plant measured with LC-MS/MS. (**B**) Tissue-specific Accumulation profiling of CGs and genin units in *C. procera* plant measured with LC-MS/MS. Each value is the mean of three replicates, and error bars indicate SDs. Asteriks *(P < 0.05) and **(P < 0.001) indicate significant differences as determined by Student’s t test. (M + H)^+^ denote parent ions of different CGs as Uzarigenin (375), Coroglaucigenin (391) Calotropagenin (405), Uscharidin (531), Calactin (533), Uscharin (586), Frugoside (537) and Ascelpin (575).

CG identification and analysis in 3 month old *C. procera* seedlings was carried out using previously established LC-MS/MS method^51^,^52^. Five CGs and three genins *viz*. Uscharidin (530), Calactin (532), Frugoside (536), Uscharin (586), Asclepin (574), Uzarigenin (374), Coroglaucigenin (390) and Calotropagenin (404) were relatively quantified based on their area peaks in 3, 6, 9, 12 month of plant (Supplementary Table 1). Genin units showed different accumulation patterns as Uzarigenin was almost equally accumulated throughout in all development stages, while maximum accumulation of Calotropagenin and Coroglaucigenin was observed in 3 month old plant seedlings (Fig. 1A). All the five CGs showed maximum accumulation in 3 month old seedlings followed by 12- month plant except for Frugoside whose accumulation was more in 9 month old plants (Fig. 1A). To further explore the metabolite accumulation pattern, tissue specific CG profiling was done in 3 month old seedling. All the CGs and their respective genin units were accumulated more in the stem tissue when compared to root and leaf tissues (Fig. 1B). However, a considerable amount of CGs were observed in the root and leaf tissues (Fig. 1B). Based on CG profiling data, 3 month old whole seedlings were selected for transcriptome sequencing and analysis.

### Transcript sequencing and its *de novo* assembly

Transcriptome sequencing of 3 month old *C. procera* whole seedling was carried out and a total of 93609100 raw reads having average length of 100 nucleotides were obtained. After stringent quality assessment and data filtering, 90598670 high quality reads with base quality greater than 30 were selected for further analysis. A comparative summary statistics of the transcriptome sequencing and assembly is shown in Table 1. *De novo* assembly of the raw reads was done using Velvet and Oases-0.2.08 assembler programs at various k-mers and it was concluded that hash length “59” (k-mer) showed better assembly in terms of total number of contigs generated, maximum contig length, total contig length and less number of N’s. Using Velvet assembler, reads were mapped into contigs set through fragmented assembly. A total of 75530165 (83.4%) of high quality reads (Hash length = 59, N50 value = 1402) were assembled by using Velvet, and 84423412 (93.2%) raw reads (Hash length = 59, N50 value = 2574) were assembled by using Oases-0.2.08. A total of 41549 contigs with average length 730.4 base pair was obtained using Velvet (Table 1), whereas Oases-0.2.08 assembled 53256 contigs with an average length of 1903.2 (Table 1). The number of contigs and their average length was more in case of Oases-0.2.08 assembler when compared to Velvet assembler. Final contigs were clustered via CD-HIT- EST to obtain 44074 singletons (unigenes) (Hash length = 59, N50 value = 2457). The majority of unigenes ranged from 1001 to 2000 base pair (Supplementary Figure S1) with an average length of 1785 base pairs. Sequences generated from the library have been deposited in National Centre for Biotechnology Information Short Read Archive (SRA) database (http://www.ncbi.nlm.nih.gov/sra/) with accession no. SRR1554320.

### Transcriptome annotation and Functional classification

**Figure 2:**
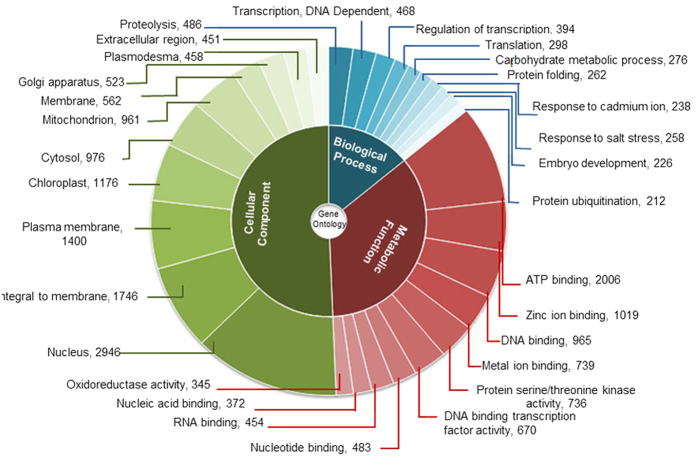
Gene ontology analysis of *C. procera* unigenes. The results are summarized in three main categories as Biological process, Molecular function and Cellular component. Slices show top ten most represented GO terms from each domain.

For the functional annotation of the assembled unigenes, sequence similarity search against known protein sequence datasets (NCBI non redundant (nr), UniPROT protein database and Pfam databases) was done using BLASTx program. The result indicated that 29356 (66.6%) unigenes had significant matches in public databases. Maximum annotation was resulted from UniPROT database while COG has annotated least number of unigenes. To further analyse the BLAST results, the E-value and similarity distribution were calculated. Statistical analysis showed that 57% of mapped sequences had significant homology (<1.0E-49) and the remaining unigenes showed homology with E-value < 1E-150 (Supplementary Figure S2A). It was observed that about 18% of annotated transcripts had a similarity higher than 80% while 47% unigenes showed similarity between 60–80% (Supplementary Figure S2B). The total 65% of transcripts showing higher identity along with high quality e-value supported reliability of *de novo* assembly performed in the study. The assembled unigenes showed maximum hits to sequences of *Arabidopsis* (49%). The next closest plant was *Thellungiella* with 37% resemblance followed by 10% with *Oryza* (Supplementary Figure S2C). Annotated unigenes of *C. procera* were functionally categorized based on Gene Ontology (GO) classification system and each unigene was allotted at least one GO term. GO annotations were further classified into three major functional ontologies as biological process, cellular component and molecular functions. Top 10 categories of each class are represented in Fig. 2. Within the Biological process category, 2490 GO terms were assigned to 31,323 unigenes. “Proteolysis” (486 unigenes), “transcription” (468 unigenes) and “regulation of transcription” (394 unigenes) were among the top three represented groups respectively. Total 21878 unigenes categorized to Molecular function category with 1513 GO terms, top three abundant groups as “ATP binding” (2006 unigenes). “Zinc ion Binding” (1019 unigenes) and “DNA binding” (965 unigenes). Cellular components category included a total of 294 GO terms with 16358 unigenes. Top three categories in Cellular components were “nuclear”, “integral to membrane” and “plasma membrane” with 2946, 1746 and 1400 unigenes respectively (Fig. 2).

### Metabolic pathway analysis by Kyoto Encyclopaedia of Genes and Genomes (KEGG)

**Figure 3:**
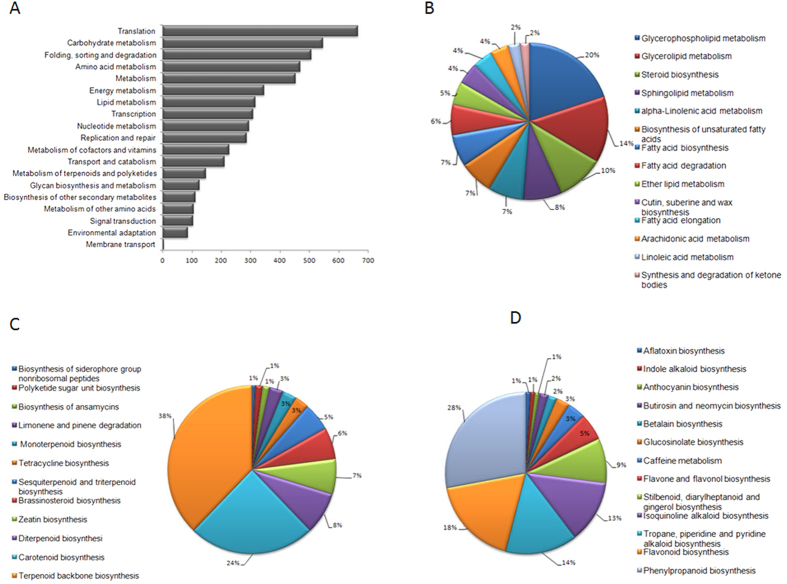
KEGG analysis of *C. procera* unigenes. (**A**) Percentage of *C. procera* unigenes in 19 sub-categories of metabolic pathway category. (**B**) 13 sub-sub categories of metabolism of terpenoids and polyketides. (**C**) 12 sub-sub categories of biosynthesis of other secondary metabolites. (**D**) 14 sub-sub category of Lipid metabolism.

Kyoto Encyclopaedia of Genes and Genomes pathway database helped us in identifying unigenes involved in metabolic pathways and their biological functions^53^. All the unigenes were analysed in the KEGG pathway database with an E-value cutoff of less than 10^−5^. A total of 5324 (12.6%) annotated unigenes showed significant matches in KEGG database and were categorized in 19 pathway functions involving 133 KEGG pathways of plant metabolism (Fig. 3A). Highest numbers of unigenes were clustered into “Translation pathway (665 unigenes)” followed by “Carbohydrate metabolism (546 unigenes)”, “Folding, sorting and degradation (506 unigenes)”, “Amino acid metabolism (468 unigenes)” and others. To further explore the function of the unigenes in relation to secondary metabolism “Metabolism of terpenoids and polyketides (148 unigenes)”, “Biosynthesis of other secondary metabolites (111 unigenes)” and “Lipid metabolism (316 unigenes)”, were further assigned to 14, 13, 12 subcategories respectively. Terpenoids backbone biosynthesis (56), Carotenoid biosynthesis (36), and Diterpenoid biosynthesis (12) were the top categories of “Metabolism of terpenoids and polyketides” (Fig. 3B). “Biosynthesis of other secondary metabolites” had Phenylpropanoid biosynthesis (31), Flavonoid biosynthesis (20) and Tropane, piperidine and pyridine alkaloid biosynthesis (16) as the top categories (Fig. 3C). Top three representatives of “Lipid metabolism” included Glycerophospholipid metabolism (63), Glycerolipid metabolism (43) and Steroid biosynthesis (31) (Fig. 3D). Unigenes involved in terpenoids, steroid and other secondary metabolite biosynthesis were well represented in the data. This information was used for the identification of genes involved in cardiac glycoside biosynthesis in *C. procera.*

### Identification of transcripts involved in cardiac glycoside biosynthesis

**Figure 4:**
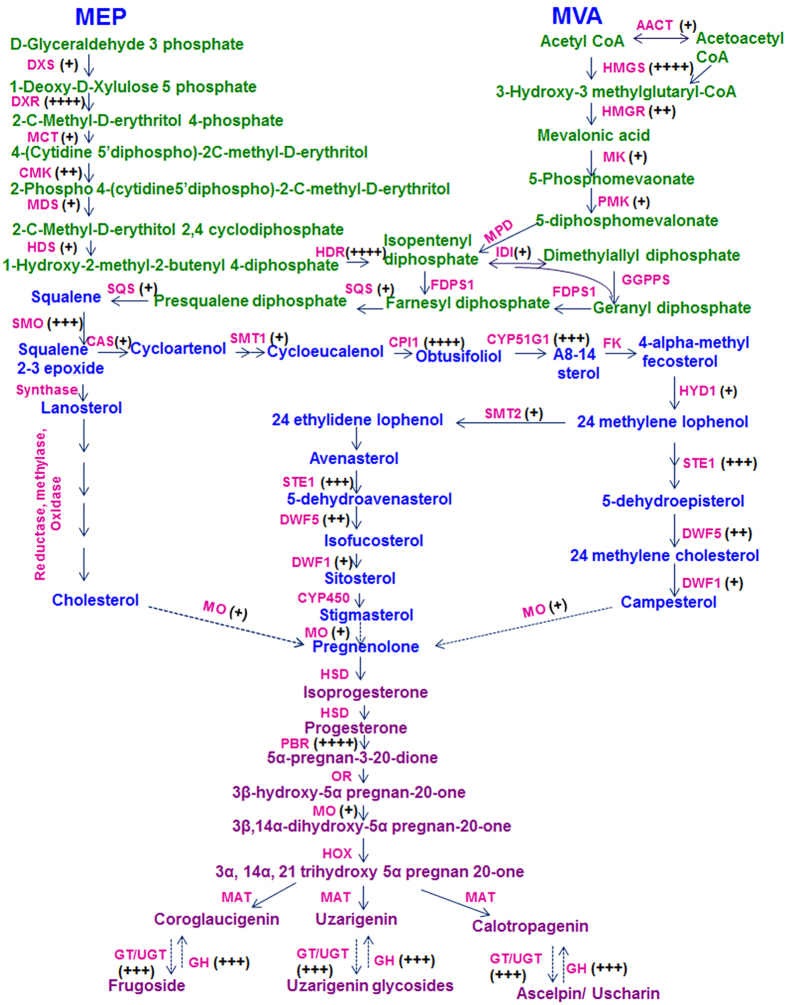
Putative cardenolide biosynthetic in *C. procera.* The pathway has been divided into three subgroups as terpenoid backbone biosynthesis, steroid biosynthesis and cardenolide biosynthesis. The enzymes with corresponding unigenes are mapped. Fold induction is represented by + sign; 0–2(+), 2–4(++), 4–10(+++), >10(++++) Full names of genes are mentioned in Supplementary Table 4.

Previous studies suggest that CGs are synthesized in plants via intermediate Pregnanes^54^. Formation of this intermediate molecule is supposed to be derived from mevalonic acid via triterpenoid and phytosterols intermediate. Predominance of either terpenoid pathway precursors (MVA or MEP pathway) in synthesis of cardenolide intermediate is not clear yet^55^,^56^. Total 47 unigenes related to terpenoid backbone biosynthesis were annotated from the transcriptome data. Together these unigenes represent 16 gene families of terpenoid backbone biosynthesis^57^. Identification of transcripts related to enzymes of both terpenoid sub-pathways suggest that precursor from both pathways are utilized in synthesis of various intermediates in *C. procera* (Fig. 4). Formation of Squalene embraces the beginning of steroid backbone which through formation of several intermediates ends up in Pregneolone^58^. These steps are catalysed by 13 steroid biosynthesis-associated gene families, mainly including reductase, oxidases and methylases, out of all 12 homologues were identified from our transcriptome data. These 12 gene families were represented by 53 unigenes in the datasets (Fig. 4).

Formation of Pregnelone is the branch point for cardiac glycoside backbone biosynthesis. Total 11 gene families are assumed to be involved in conversion of Pregenolone to cardiac glycoside out of which, only five gene families were identified in the study. Conversion of Pregnenolone to Progesterone is catalysed by *3β-HSD* enzyme^59^. In our study, 15 transcripts were showing homology to *3β-HSD,* but all were partial sequences none coding for full ORF. Once the progesterone is synthesized it is immediately reduced to one of intermediates Pregnane-dione with the enzyme *5βPOR*^60^,^61^. Full length gene coding for *5βPOR* was annotated from two transcripts. Four transcripts showing similarities with glucohydrolase enzymes were identified which may have *cardenolide 16-O-glucohydrolase* enzymes like activity. Till date only one gene, i.e. *5βPOR, progesterone 5β-reductase* have been characterised from this plant and our transcript annotating to *5βPOR* showed 100% homology to this gene sequence^62^.

*Cytochrome P450 monoxygenase* are mediators of oxidation reaction involved in the synthesis of large variety of secondary metabolites in plants^63^. Total 90 transcripts were annotated to different monoxygenase which may be involved in various steps of cardiac glycosides biosynthesis. Similarly 125 transcripts were annotated to *GT*/*UGT (glycosyl transferase*/*glucuronosyl transferase*/*UDP-glycosyl transferase*/*glucuronosyl transferase*) which may be involved in the last steps of cardiac glycosides biosynthesis. They catalyse glycosylation of genin units to glycosides. Identification of the specific GT/UGT, *Mono-oxygenase* and *Glucohydrolase* involved in biosynthesis and inter-conversion of genin and glycoside will impart a new direction towards the study of the cardiac glycoside biosynthetic pathway and its related genes. (Fig. 4)

### Tissue specific expression profiling of transcripts involved in cardiac glycoside biosynthesis

**Figure 5:**
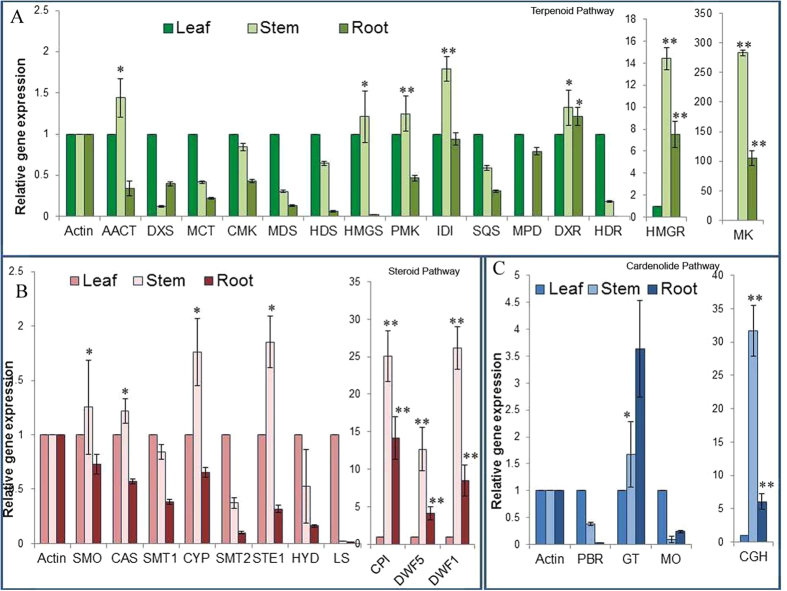
(**A**) Tissue specific expression of genes involved in terpenoid backbone biosynthesis by qRT-PCR. (**B**) Tissue specific expression of genes involved in steroid backbone biosynthesis by qRT-PCR. (**C**) Tissue specific expression of genes involved in cardenolide biosynthesis by qRT-PCR. The stem and root group compared with leaf group. The data represents three independent biological and experimental repeats with standard deviations. Asterisk *(P < 0.05) and **(P < 0.001) indicate significant differences as determined by Student’s t test. Full names of genes are mentioned in Supplementary Table 4.

To lay down the biosynthetic pathway and correlation of the CGs accumulation with the putative transcripts which were identified in the study, tissue specific expression of thirty transcripts representing different gene families involved in CG biosynthesis were studied (Fig. 5).

Almost all the transcripts were accumulated in the aerial part (leaf and stem) of the seedling. Most transcripts of terpenoidal pathway were showing accumulation in aerial parts (leaf and stem). Out of 15 putative genes studied, 07 were significantly up-regulated in the stem when compared with the leaf tissue. Maximum accumulation was observed for *Mevalonate Kinase (MK*) and *hydroxymethylglutaryl-CoA reductase (HMGR*) in stem (Fig. 5A). Similarly majority of transcripts, i.e. 07 out of 11, coding genes for steroid backbone were significantly up-regulated in the stem when compared with leaf and root tissue. Maximum accumulation was observed for transcripts of *Cyclopropyl isomerase (CPI*), *Delta 24-sterol reductase (DWF1*) and *Sterol delta 7 reductase (DWF5*) (Fig. 5B). Among five studied transcripts of cardiac glycosides pathway studied, maximum accumulation of about 31.7 fold in stem was observed for *Cardenolide glucohydrolase (CGH*) (Fig. 5C).

Tissue specific expression of transcripts involved in CG biosynthesis was in accordance with the CG accumulation in different tissues where most of cardiac glycosides were observed in the aerial parts of the seedlings particularly stem tissue.

### Effect of wound treatment on cardiac glycoside accumulation and their related biosynthetic genes

**Figure 6:**
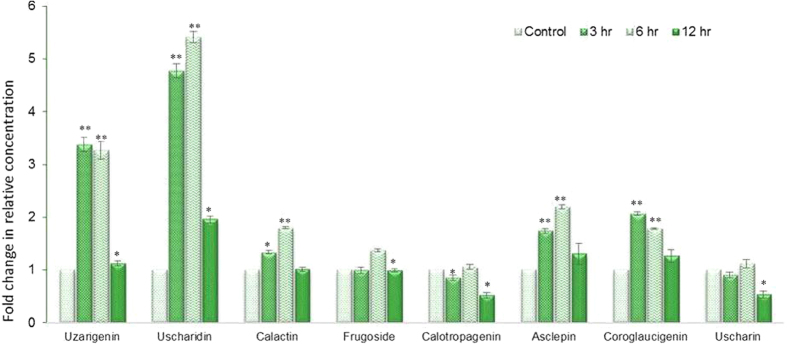
Accumulation of CGs and genin units after 3, 6, 12 hr of wounding in *C. procera* whole plant measured with LC-MS/MS. Each value is the mean of three replicates, and error bars indicate SDs. Asterisk *(P < 0.05) and **(P < 0.001) indicate significant differences as determined by Student’s t test.

**Figure 7:**
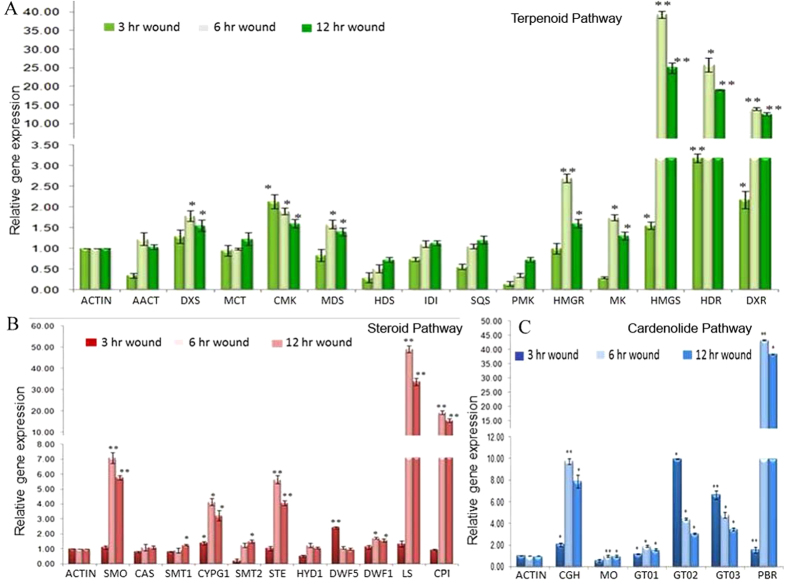
Relative expression level of genes involved in cardenolide biosynthesis after wound treatment. (**A**) Effect of wound on expression of genes involved in terpenoid backbone biosynthesis by qRT-PCR. (**B**) Effect of wound on expression of genes involved in steroid backbone biosynthesis by qRT-PCR. (**C**) Effect of wound on expression of genes involved in cardenolide biosynthesis by qRT-PCR. Actin was used as internal reference gene and relative abundance of each gene was compared after 3, 6 and 12 hr of wounding. The data represents three independent biological and experimental repeats with standard deviations. *(P < 0.05) and **(P < 0.001) indicate significant difference in gene expression (control vs wound). Full names of genes are mentioned in Supplementary Table 4.

Many secondary metabolites found in plants have a role in defence against herbivores, pest and pathogens^64^. Several classes of these secondary products are induced by infection, wounding or herbivore^65^. To study the effect of wounding on accumulation of cardiac glycosides we subjected 3 month whole seedlings to mechanical wounding for 3, 6 and 12 hours and estimated cardiac glycosides using LC/MS-MS. Five out of seven metabolites showed significant accumulation in response to wound stress. Genin units were accumulated maximally in response to 3 hr of wounding and maintained higher concentration till 6 hr and reverted to their basal level within 12 hours of stress (Fig. 6). Uzarigenin showed more than 3-fold induction in 3 and 6 hr of wounding, two-fold accumulation in Coroglaucigenin concentrations were observed in response to 3 hr of wounding. Level of Calotropagenin remained unaltered after wounding (Fig. 6). Cardiac glycosides showed different accumulation patterns as their level increased up to 6 hr of wounding and then returned to the basal level. Highest accumulation was observed for Uscharidin which was up to 4.8 and 5.4 folds higher in comparison to control after 3 and 6 hr of wounding respectively (Fig. 6). Similarly, Calactin and Asclepin also showed 1.8 and 2.2-fold accumulation after 6 hr of wounding when compared with control whereas a significant increase of 1.4 fold was observed in Frugoside concentration after 6 hr of wounding (Fig. 6). There was no increase in level of Uscharin concentration up to 6 hr of wounding and after 12 hr of wound stress the level of Uscharin decreased significantly as compare to control (Fig. 6). To correlate the accumulation pattern of cardiac glycosides with 32-putative genes of biosynthetic pathway, a relative expression analysis was done after 3, 6, 12 hr of wound treatment. Like metabolites almost three-fourth of putative genes were also significantly accumulated in response to wound stress. Among 15 transcripts of terpenoid pathway, 8 showed induction in the range of 1.5–40 folds. Majority of them showed gradual increase in the transcript level at 3 hr of wounding which reached maximum at 6 hr followed by decrease in the accumulation of transcripts after 12 hr of wounding. Three transcripts *viz hydroxymethylglutaryl-CoA (HMGS*), *4-hydroxy-3-methylbut-2-enyl diphosphate reductase (HDR*) *and 1-Deoxy-D-xylulose-5-phosphate reductoisomerase (DXR*) showed high accumulation of 40, 25 and 15 folds respectively after 6 hr of wound stress (Fig. 7A). There was no significant effect of wounding on transcripts of *Acetyl-Co-AC-acetyltransferse (AACT*), *2-C-methyl-D-erythritol4-phosphate cytidylyltransferase (MCT*), *4-hydroxy-3-methyl-2-en-1-yldiphophate synthase (HDS*)*, Isopentenyl-diphosphate delta-isomerase (IDI*)*, Squalene synthase (SQS*) *and Phosphomevalonate Kinase (PMK*).

Similarly, transcripts identified in steroid biosynthesis were also found to be wound responsive except for *SMT1, SMT2, CAS* and *HYD1*. All induced transcripts uniformly showed highest accumulation at 6 hr of wounding except for *Sterol reductase (DWF5*). *Lupeol synthase (LS*) showed maximum accumulation of 49-fold followed by 19-fold induction of *CPI* after 6 hr wounding. *Squalene monoxygenase (SMO*)*, Sterol desaturatses (STE1*)*, CYP51G1* and *Sterol reductase (DWF1*) were also significantly up-regulated in the range of 1.7–7 folds after 6 hr of wound stress (Fig. 7B). Among the genes of the cardenolide pathway most of the transcripts were significantly induced upon wound treatment. Highest accumulation of >43-fold was observed for *Progesterone 5β-reductase (5βPOR*), a key enzyme of CG biosynthesis followed by >9-fold induction of *Cardenolide glucohydrolase (CGH*) another important enzyme of the pathway (Fig. 7C). Last steps of the cardiac glycosides biosynthesis include glycosylation of respective genin units to their glycoside which are catalysed by *Glycosyltransferases* (GT). All the three GT transcripts studied were wound responsive, but the maximum accumulation of >10 fold was observed for GT*02* in response to 3 hr of wounding, There was no alternation in the expression of selected *mono-oxygenase* transcript in response to wounding.

## Discussion

With establishment of economic importance of *C. procera*, in the last five years this plant has been explored for the identification of genes involved in stress biology^66^,^67^, genes related to fibre development^68^ and profiling of Cysteine Protease from this plant^69^ through *de novo* sequencing. Despite of immense medicinal importance of Cardiac glycosides of this plant, putative genes involved in biosynthesis of CG have not been studied well. *De novo* transcriptome sequencing proves a promising tool for gene identification, especially for those plants whose genome sequences are unavailable^70^,^71^. Using similar approach putative biosynthetic pathways have been identified from important medicinal plants^72^,^73^. One such approach was utilized in case of *Digitalis purpurea* for identification and elucidation of putative CG biosynthetic pathway^74^.

Stage specific CGs accumulation suggested that young plants (3 month old) are better candidate for transcriptome analysis as they accumulate more CGs in comparison to mature plants. These findings were in agreement with pervious finding for CG accumulation in different plant^75^. Secondary metabolites are synthesized as per specific requirements of plants in response to the nature, the probable cause of more CGs biosynthesis in young plants (3 month old) may be due to defence against wounding or herbivore as young plants are more prone to it^76^. From tissue specific metabolic profiling it was concluded that although, maximum accumulation was in the stem, but the considerable amount of CGs was detected in the other tissues also. So, three month whole seedlings were selected for the transcriptome sequencing to elucidate the cardiac glycoside biosynthetic pathway.

*De novo* assembly of *C. procera* resulted in 44074 unigenes with an average length of 1785 base pairs. More than 66% assembled sequenced were annotated, out of which 75% of the annotated sequences showed high sequence similarities (>60%) with the known protein from different databases further validating the sequence assembly. The annotation and similarity percentage was in accordance with the previous findings^77^,^78^. A total of 4297 GO terms were categorised under ‘biological processes’, ‘molecular function’ and ‘cellular component’. The rest of the sequences probably remained unannotated due to the un-translated regions/non coding RNA or short sequences not having any conserved protein domain, as well assembly mistakes cannot be ruled out. Mapping of unigenes onto the KEGG pathways helped in the identification of a large number of unigenes involved in biosynthesis of various metabolites in *C. procera*.

Cardiac glycosides are synthesized in plants via Pregnanes, which utilise a number of intermediate from terpenoidal and steroidal pathways^79^. About 100 unigenes, representing 29 gene families from both these sub-pathways were identified from the data set. Pregnane undergoes various modifications to yield final products as cardiac glycosides which involve 11 gene families. 236 unigenes, representing transcripts of *Δ5-3, β-hydroxysteroid dehydrogenases/3-KSI*, 5-3-ketosteroid isomerase (in total as 3β-HSD), *progesterone 5β-reductases (5β-POR*), *mono-oxygenases, cardenolide 16′-O glucohydrolases (CGH*), *and glycosyltransferases (GT*) were identified. 5β-POR is the only enzyme characterized from *C.procera.* It catalyse the transformation of progesterone to 5β-pregnane-3, 20-dione.

Fifth most abundant transcript of the data set belongs to Mono-oxygenase with 90 transcripts representing 40 loci; shedding light on the active steroid biosynthesis within the plant. Glycosylation is the last step in cardiac glycosides biosynthesis which is catalysed by a super large gene family in plants UGTs^80^. These are key modifying enzymes in the CG biosynthesis and are largely responsible for its diversity within plant *Calotropis*. Large numbers of GT/UGTs are required for the synthesis of diverse type of CGs within plant as represented by the large number of unigenes with the same function^81^. Also 04 loci of Cardenolide glucohydrolases enzymes involved in hydrolysis of primary CGs have been identified from the data set. The representative transcripts associated with biosynthesis were accumulated more in stem tissues followed by leaf and roots in 3 month old plants, explaining that probably in young plants, green stem and leaves are site of CG biosynthesis and its accumulation. Our results showed parallel accumulation of cardiac glycosides and transcripts related to CG biosynthesis in a tissue specific manner.

Cardiac glycosides have been evolved as a means of the general defence system^82^ and are widely distributed among different genera of Apocynaceae^83^,^84^,^85^. Various elicitations and mechanical wounding (mimicking herbivory) have been shown to enhance these chemical defence of plants. These finding paved the way to study the effect of wounding on accumulation of CGs and we observed that CGs get accumulated in response to wound stress. Similar induction in level of CGs were recorded after mechanical damage over a short period of time in *Asclepias*^86^. Enhanced cardenolide accumulation after application of mechanical wounding has also been reported in *Calotropis gigantea* hairy root culture^87^. In our study a similar induction in level of cardiac glycosides was observed after wound treatment. Genin units which are storage forms of cardiac glycosides showed early response to wounding. Wound treatment might be inducing their secretion from vacuoles so that they may be readily available in free form to get converted into their respective glycosides. Respective glycosides like Uscharidin, Asclepin, Calactin were also wound responsive but a late induction pattern (at 6 hrs) was observed for them. Wound may also be stimulating translocation of already synthesised cardiac glycosides from their site of synthesis to site of action which explains their late induction at 6 hrs.

It has been observed that terpenoid^88^ and cardiac glycosides gets accumulated on wounding stress^89^, therefore, identified transcript were studied for their accumulation in response to wound stress and it was observed that most of the transcript were wound responsive and were accumulated in different patterns. HMGR and DXS are rate limiting steps of isoprenoid pathway and their transcript level increases upon wounding^90^, the same was observed in our study. It was observed that increased expression of *5β*–*POR* was consistent with higher accumulation of CGs in *Digitalis* plant^91^. In our study we also observed the same co-relation between higher accumulation of CGs and *5β*–*POR*. Early increase in transcript level of GT may be explained by the fact that upon wounding genin gets rapidly glycosylated and convert into glycosides for immediate defence response. 64 unigenes were annotated as GT and only three were studied for wound response. There exists possibility of more wound responsive GT which may be playing an active and specific role in cardiac glycosides biosynthesis. Identification of such genes will further elaborate the facts on biosynthetic pathway in *Calotropis procera*. Increased expression of most of the transcripts in response to wounding suggested that CGs are probably synthesized upon wounding and it may have some role in wound response or wound mediated signal transduction within plants. The present study has helped in identification of novel cardiac glycosides related transcripts involved in CG’s biosynthetic pathway. Such approach of wound treatment to induce and identify metabolic pathway genes has been used in number of plants like *Catahranthus*^92^, *Nicotiana*^93^ and *Opium*^94^. The characterization of these gene transcripts in either homologous or heterologous system will further lay down the progress towards metabolic engineering of CG’s. However, such metabolic engineering requires complete knowledge of wound response of a particular metabolite as well as associated biosynthetic genes.

## Method

### Plant Material and Wound treatment

Seeds of *C. procera* were planted and grown in pots filled with a mixture of soilrite and vermiculite (1:3, v/v) for 3, 6, 9 and 12 months. The plants were watered every alternate day. Whole plant was harvested at respective months and dried in oven for LC/MS analysis. Root, stem and leaf were also harvested at 3 month old plant. For gene expression analysis, whole seedling was washed with DEPC treated water, dried, frozen in liquid nitrogen and stored at −80 °C for further analysis. For wound treatment, 3 months old seedling was pricked with sterile needle uniformly and harvested after 3 and 6 and 12 hr respectively.

### cDNA library construction for *de novo* Sequencing

Tissues from 10 seedlings (3 month old whole seedling) were pooled together and RNA was isolated using Trizol reagent (Invitrogen Corp., Carlsbad, CA) and subsequently used to construct library with TruSeq RNA Sample preparation kit V2 (Illumina, Cat RS-122-2002) following manufactures protocol. 1 μg of total RNA by Qubit was enriched for Poly A using RNA Purification Beads. Enriched Poly-A RNA was fragmented for 4 minutes at 94 °C in the presence of divalent cations. First strand cDNA was made by Superscript III reverse transcriptase (Invitrogen, USA) using random hexamers followed by synthesis of second strand cDNA in the presence of DNA polymerase I and RNaseH. The cDNA was cleaned using Agencourt Ampure XP SPRI beads (Beckman Coulter, USA). SPRI cleaned cDNA was ligated with “Illumina Adapters” after end repair and addition of “A”- base. For the enrichment of adapter ligated fragments, library was amplified using 8 cycles of PCR. The prepared library was quantified using nanodrop. Quality validation of the library was done by running an aliquot on a High Sensitivity Bioanalyzer Chip (Agilent Technologies, Palo Alto, CA, USA).

### Sequencing and *de novo* assembly

Illumina HiSeq was used to generate 2x Paired end (101 bp max) raw reads. Quality control of Illumina Hi Seq paired raw reads was done using SeqQC V2.1. In-house script was used for low quality bases trimming towards 3′-end. Adapter sequences and empty reads were also trimmed before assembly of reads. *de-novo* assembly of Illumina HiSeq data was performed using velvet-1.2.10^95^ for various k-mers and it was concluded that the hash length (k-mer) 59 was better than others considering various parameters like total number of contigs generated, maximum contig length, total contig length and less number of N’s.

Using Velvet assemblies as input, transcriptome assembly was done by Oases 0.2.08 with hash length 59, under default parameters with minimum transcript length cutoff of 20 bp^96^. These Velvet assemblies were grouped into loci which were later used for construction of transcript isoform. Transcripts with at least 200 bases were considered for further analysis. Unigenes were further processed to remove redundant sequences. Using CD-HIT v4.5.4, clustering of unigenes was done and non-redundant unigenes were acquired^97^.

### Sequence annotation and Functional classification

Annotation of unigenes was done using the BlastX algorithm^98^. Various protein databases like NCBI non-redundant protein database^99^, UniProt^100^, Pfam^101^ were searched for homologs. The Blast algorithm was used to identify homologous sequences with a cut-off value less than 10^−5^. Gene name were assigned to each sequence based on best Blast hit. GO annotation was assigned to the all the annotated unigenes using Blast2GO program^102^. GO terms were mainly divided into biological processes, molecular functions and cellular components. Assembled sequences were assigned (KEGG) pathways using online KAAS (KEGG Automatic Annotation Server). KEGG-orthology (KO id) was procured through bi-directional best hit method^103^.

### Sample preparation and analysis of CGs by Liquid chromatography/ Mass spectrometry

Plant parts harvested was dried in oven at 37 °C. These samples were grinded to fine powder with the help of mortar and pestles. 500 mg of each sample was extracted with 10 ml absolute ethanol and kept on shaker for 48 hr, after which extract were filtered by Whatmann No. 1 filter paper and filter sterile through 0.25 micron filters. The LC-MS and LC-MS/MS were performed on a Waters TQD triple quadrupole mass spectrometer (USA). It was equipped with waters, H-Class Acquity UPLC system and electrospray ionization source. The UPLC column used was water BEH C-18 100 × 2.1 mm, 1.7 μm and dual mode (±) LC-ESI-MS experiments performed after injecting 1 μl samples by the autosampler. Similarly, LC-ESI-HRMS analysis was performed on Agilent 6500 Q-Tof Mass Spectrometer (USA). It was equipped with electrospray ionization (ESI) interface using the following operation parameters: capillary voltage 3.5 kV, nebuliser 40 psi, drying gas (nitrogen) flow rate 11.0 L/min, drying gas temperature 300 °C, fragmentor 150 V, skimmer voltage 65 V. Mass spectra were recorded across the range *m*/*z* 150–1000 Th. The accurate masses of selected metabolites were observed and compared with their exact mass (theoretical mass) values manually. Analyses were performed as per our previously established method. *m/z* values and retention time of different compounds identified are shown in Table 2.

### Quantitative Real-Time PCR analysis

For qRT-PCR RNA from 3- month old seedlings isolated using Trizol reagent and quantified using Nanodrop. 5 μg total RNA from the samples was taken for making single-stranded cDNA using High Capacity cDNA Reverse Transcription Kit (Applied Biosystems, USA). cDNA was diluted 10 times with de-ionised water. Primers used in the study are given in supplementary table 3. The reaction was performed on 7500 FAST Real Time PCR system using SYBR Premix Ex Taq (Takara). Melting curve analysis was done to verify the reaction specificity. Following thermal cycling parameters were used: initial denaturation (95 °C for 5 min); 40 amplification cycles (95 °C for 20 sec, 58 °C for 30 sec, 72 °C for 20 sec). Internal reference gene Actin was used to normalise expression levels of unigenes. Relative expression level of unigenes in tissues was calculated using the delta-delta Ct (2−ΔΔCt) method^104^. All the experiments were expressed as mean ± SD after normalization.

## Conclusion

With critical observation and the best of our knowledge, we found that 3 months old seedlings had maximum CGs accumulation which was validated through Cardiac Glycoside profiling at different developmental stages of *C. procera*. For the first time Illumina HiSeq 2x Paired end method was used for the transcriptome assembly and characterization of *C. procera* (3 months old seedling was used). This work represents the *de novo* transcriptome sequencing analysis of tissues of 10 whole plant seedling RNAs. Total 44074 unigenes were generated, and 5324 unigenes were matched with KEGG database, which are involved in 133 different pathways of plant metabolism. Out of 5324 unigenes, 336 unigenes were classified in 33 gene families, which were involved in CGs biosynthesis. There are a broad class of enzymes such as *Mono-oxygenases*, *Oxidoreductases*, *Tr*an*sferases*, *Hydrolases etc.* Through GO annotation and expression profiling validation, we were succeeded in sorting of the unigenes which may lead to CGs biosynthesis. Additionally, this study generated raw data which can be used for the advancement in the field of molecular markers (through Expressed sequence tag simple sequence repeats “EST-SSRs”). MIcroSAtellite identification (MISA) was the tool which can be used to identify EST-SSR repeats. This study sets a platform of valuable gene sequences for the *de novo* gene discoveries and further exploration of the CGs pathway of *C. procera*.

## Additional Information

**How to cite this article**: Pandey, A. *et al.* Transcriptome and Metabolite analysis reveal candidate genes of the cardiac glycoside biosynthetic pathway from *Calotropis procera. Sci. Rep.* **6**, 34464; doi: 10.1038/srep34464 (2016).

## Supplementary Material

Supplementary Information

## Figures and Tables

**Table 1:** Transcriptome *de novo* Assembly Statistics of *Calotropis procera.*

Sample Name	*C. procera*		
Tool used	Velvet	Oases	Cd-hit-est
Hash length	59		
Contigs Generated	41549	53256	44074
Maximum Contig Length	13530	15838	15838
Minimum Contig Length	117	200	200
Average Contig Length	730.4	1903.2	1785
Total Contigs Length	30348668	101357782	78672233
Total Number of Non-ATGC Characters	33422	87231	72447
Percentage of Non-ATGC Characters	0.11	0.086	0.092
Contigs > 100 b	41549	53256	44074
Contigs > 200 b	29565	53256	44074
Contigs > 500 b	17854	45454	36672
Contigs > 1 Kb	10033	37786	29917
Contigs > 10 Kb	7	73	45
N50 value	1402	2574	2457
No. of Reads Used	75530165	84423412	84423412
Total no. Of reads	90598670	90598670	90598670
% of reads assembled	83.36785	93.18	93.18

**Table 2:** Identification of cardiac glycosides by their characteristic fragment ions.

S. No.	RT	M.W.	Identified	*m*/*z* (Characteristic fragment ions of genin)	Genin unit	Sugar unit
01	5.34	404	Calotropagenin	387,369,351,341,333,323,305	A	—
02	6.78	536	Frugoside	391,373,355,339,337,325,293	C	146
03	6.89	390	Coroglaucigenin	373,355,339,337,325,293	C	—
04	7.85	532	Calotropin	387,369,359,351,341,333,323	A	146
05	9.04	374	Uzarigenin	357,339,321,293	B	—
06	9.46	574	Asclepin	387,369,351,341,333,323	A	188
07	9.47	530	Uscharidin	387,369,351,333,323	A	144
08	9.90	586	Uscharin	323,333,341,351	A	201

A = Calotropagenin, B = Uzaigenin, C = Coroglaucigenin [Structure characterization of all above cardiac glycosides were based on their mass spectrometry based evidence (molecular weights, accurate mass measurement/chemical formula, characteristic MS/MS fragmentation of genin unit) and literature support].
