# Supplementary material for: Transcriptome and Metabolite analysis reveal candidate genes of the cardiac glycoside biosynthetic pathway from Calotropis procera
Source: Sci Rep. 2016 Oct 5;6:34464. doi: 10.1038/srep34464 (PMC5050527; doi:10.1038/srep34464)
Supplement: Supplementary Information [file srep34464-s1.pdf]

Transcriptome and Metabolite analysis reveal candidate genes of cardiac glycoside biosynthetic pathway from *Calotropis procera*

Akansha Pandey[1], Vishakha Swarnkar[1#], Tushar Pandey[1#], Piush Srivastava[1], Sanjeev Kanojiya [2], DK Mishra[1], Vineeta Tripathi[1*]

[1]Botany division,CSIR-CDRI,_ Sector 10, Jankipuram Extension, Sitapur Road, Lucknow 226031, Uttar Pradesh, INDIA, [2] Sophisticated Analytical Instrument ,CSIR-CDRI, Sector 10, Jankipuram Extension, Sitapur Road, Lucknow 226031, Uttar Pradesh, INDIA

#authors contributed equally

Supplementary Figure S1. Length distribution of contigs assembled in *C. procera*. The number of 12 groups of unigenes with different length is shown.

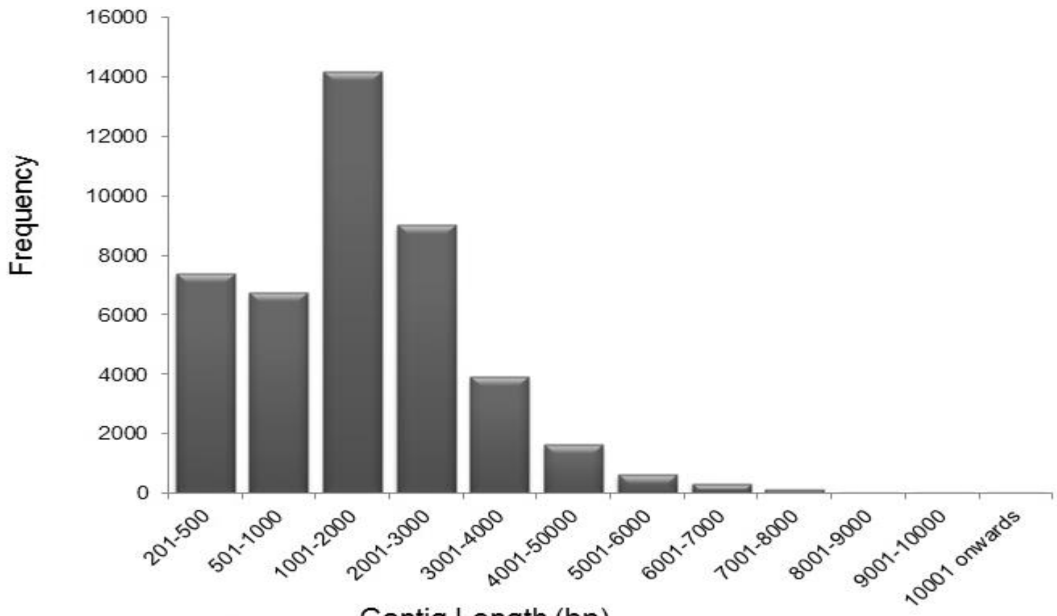

Supplementary Fig S1

Supplementary Figure S2. Characterstic of homolgy search of assembled unigenes. A-E-value distribution of top Blast hits. B- Similarity distribution of unigenes. C-Species distribution of Blast hits.

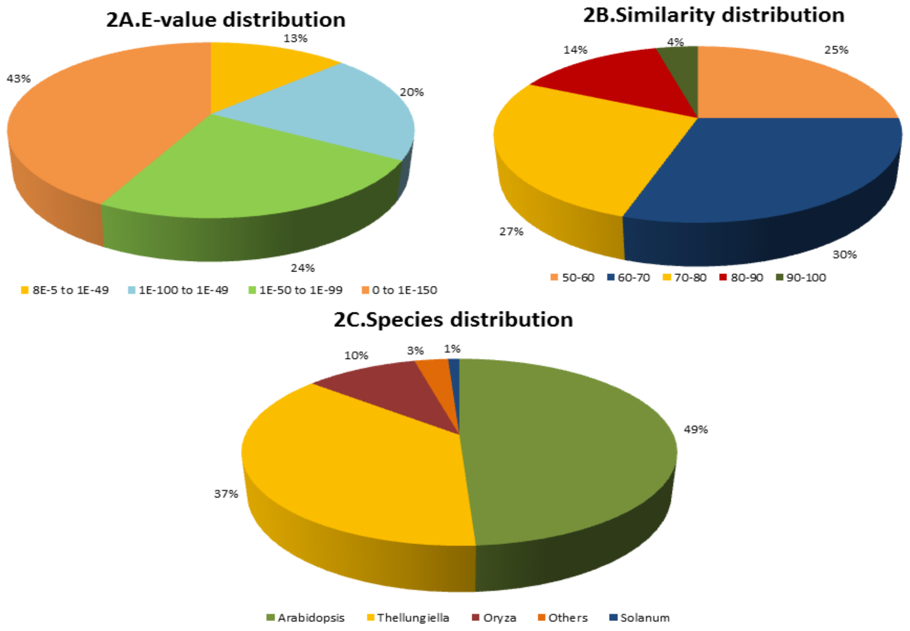

Supplementary Figure S3: Structure and spectra chromatogram of cardiac glycosides and genin units with their proposed fragmentation pattern.

S. No.	RT	M.W.	Identified	*m/z* (Characteristic fragment ions of genin unit)	Genin unit	Sugar unit
01	5.34	404	Calotropagenin	387,369,351,341,333,323,305	A	-

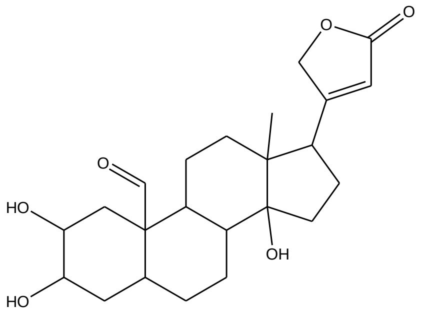

Calotropagenin
Chemical Formula: $C_{23}H_{32}O_6$
Exact Mass: 404.2199
Molecular Weight: 404.4966

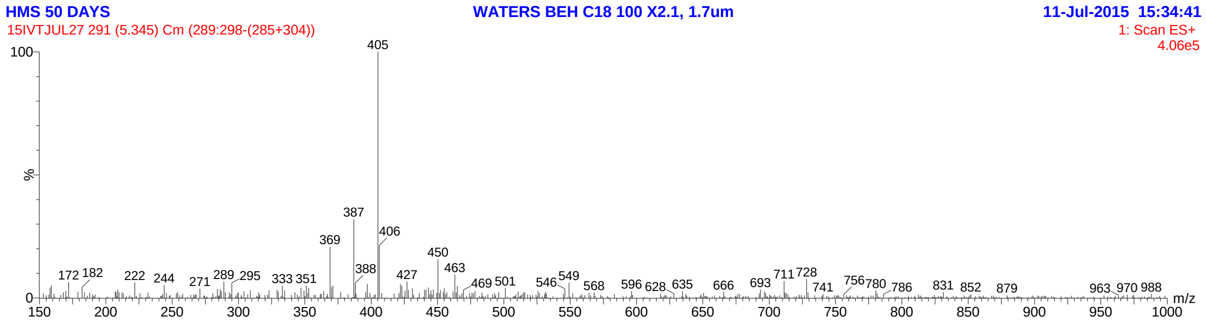

LC-ESI-MS Spectrum of Calotropagenin *m/z* 405 $[M+H]^+$

m/z	Annotation	Mass difference	Confirmation remark
405	$[M+H]^+$	1Da	Molecular weight 404
427	$[M+Na]^+$	23 Da	Da

Accurate mass observed	Annotation	Calc. mass of Calotropagenin $[M+H]^+$	Mass difference In mmu	Confirmation remark
405.2270	$[M+H]^+$	405.2272	0.0002	Molecular formula of the compounds $C_{23}H_{33}O_6^+$

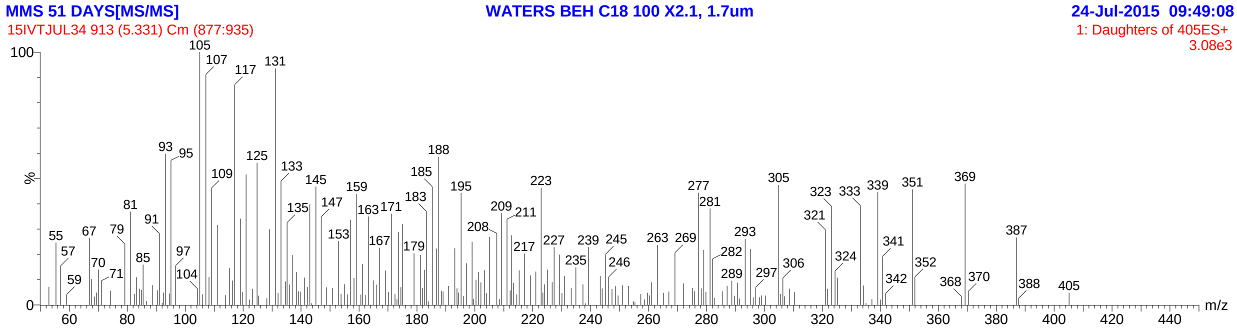

LC-ESI-MS/MS Spectrum of Calotropagenin *m/z* 405 $[M+H]^+$ recorded at 30-20ev collision energy

Reference LC-ESI-MS/MS Spectrum (Phytochem Anal, 2012. 23(2): p. 117-25)

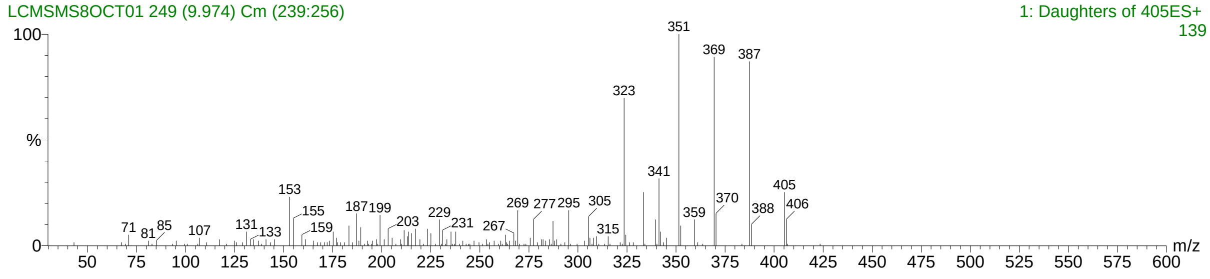

Previously published LC-ESI-MS/MS Spectrum of Calotropagenin *m/z* 405 $[M+H]^+$ recorded at 15-5ev collision energy

m/z	Annotation	Neutral losses in Da
387	$[M+H-H_2O]^+$	18
369	$[M+H-2H_2O]^+$	36
359	$[M+H-H_2O+CO]^+$	46
351	$[M+H-3H_2O]^+$	54
341	$[M+H-2H_2O+CO]^+$	64
333	$[M+H-4H_2O]^+$	72
323	$[M+H-3H_2O+CO]^+$	82

All the above neutral losses can be predicted from structure of calotropagenin as proposed in fragmentation pattern.

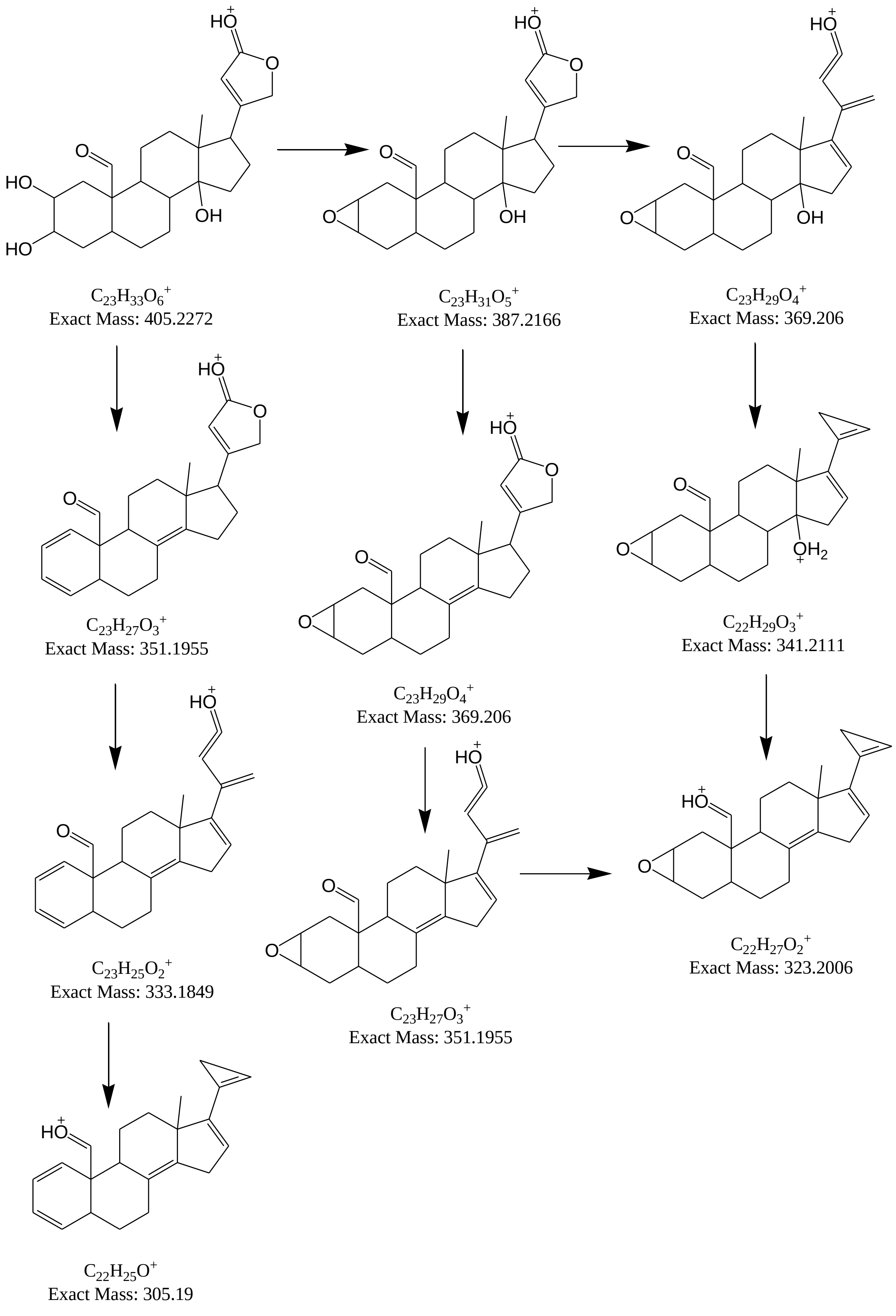

Proposed fragmentation pattern of Calotropagenin *m/z* 405 $[M+H]^+$

S. No.	RT	M.W.	Identified	*m/z* (Characteristic fragment ions of genin unit)	Genin unit	Sugar unit
02	6.78	536	Frugoside	391,373,355,339,337,325	C	146

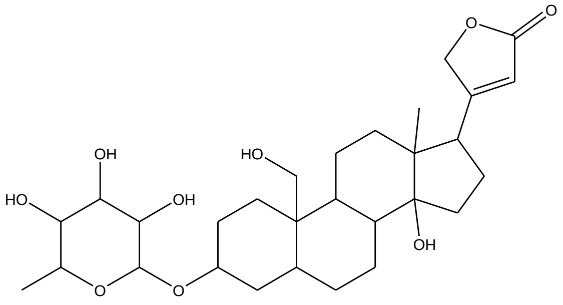

Frugoside
Chemical Formula: $C_{29}H_{44}O_9$
Exact Mass: 536.2985
Molecular Weight: 536.6543

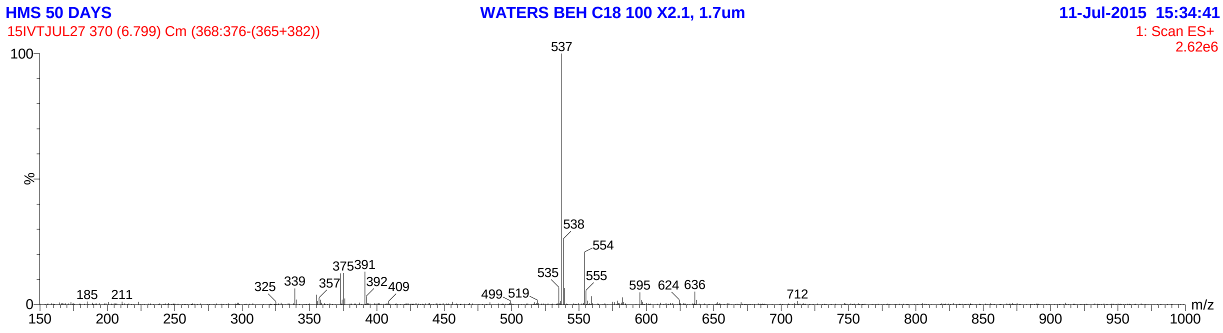

LC-ESI-MS Spectrum of frugoside *m/z* 537 $[M+H]^+$

m/z	Annotation	Mass difference	Confirmation remark
537	$[M+H]^+$	1Da	Molecular weight 536 Da
554	$[M+NH_4]^+$	18 Da	
391	$[M+H\text{-glycone}]^+$	146 Da	Cleavage of –O-glycoside bond

Accurate mass observed	Annotation	Calculated mass of Frugoside $[M+H]^+$	Mass difference In mmu	Confirmation remark
537.3057	$[M+H]^+$	537.3058	0.0001	Molecular formula of the compounds $C_{29}H_{45}O_9^+$

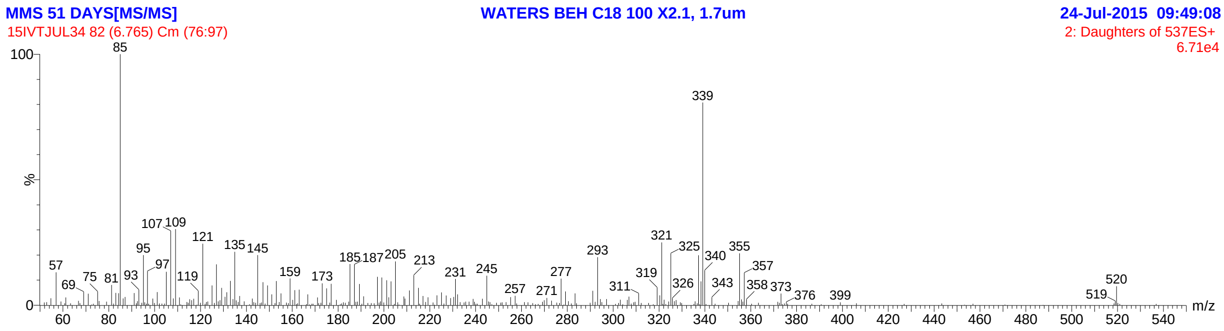

LC-ESI-MS/MS Spectrum of frugoside *m/z* 537 $[M+H]^+$ recorded at 30-20ev collision energy [characteristic fragment ions from genin observed *m/z* 373,355,339/337, 325]

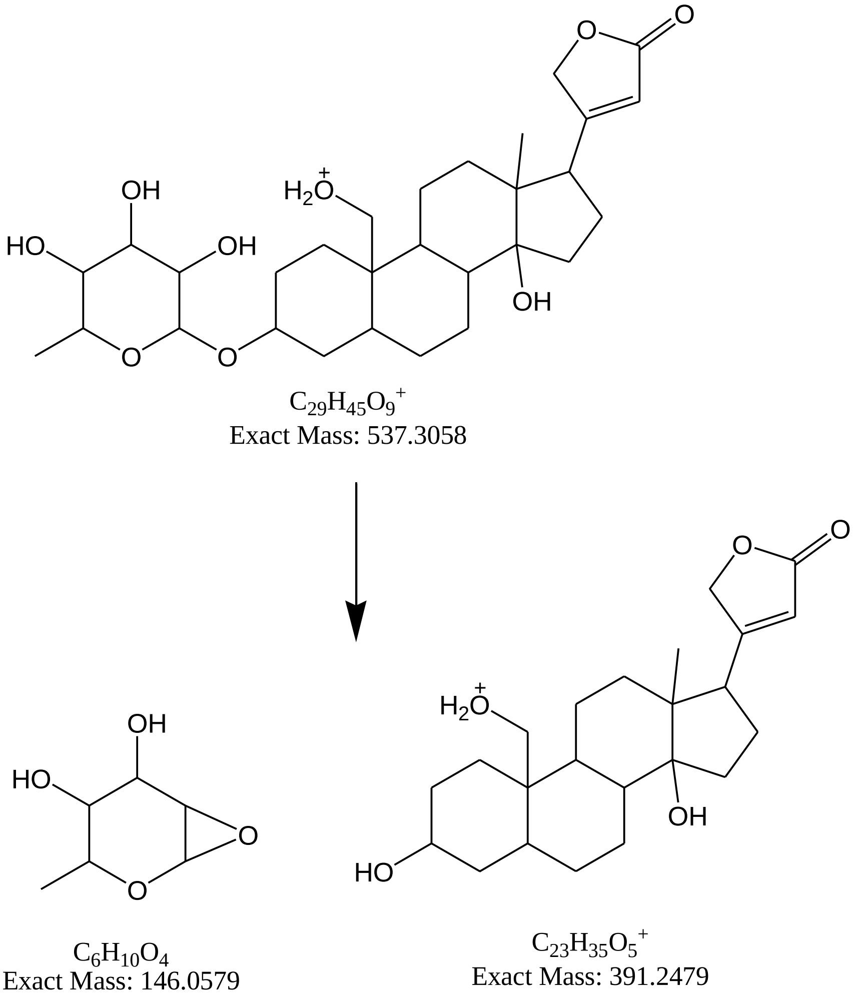

Proposed fragmentation pattern of Frugoside m/z 537 $[M+H]^+$

S. No.	RT	M.W.	Identified	*m/z* (Characteristic fragment ions of genin unit)	Genin unit	Sugar unit
03	6.89	390	Coroglaucigenin	373,355,337, 325	C	-

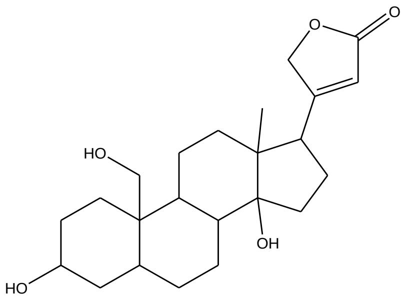

Coroglaucigenin
Chemical Formula: $C_{23}H_{34}O_5$
Exact Mass: 390.2406
Molecular Weight: 390.5131

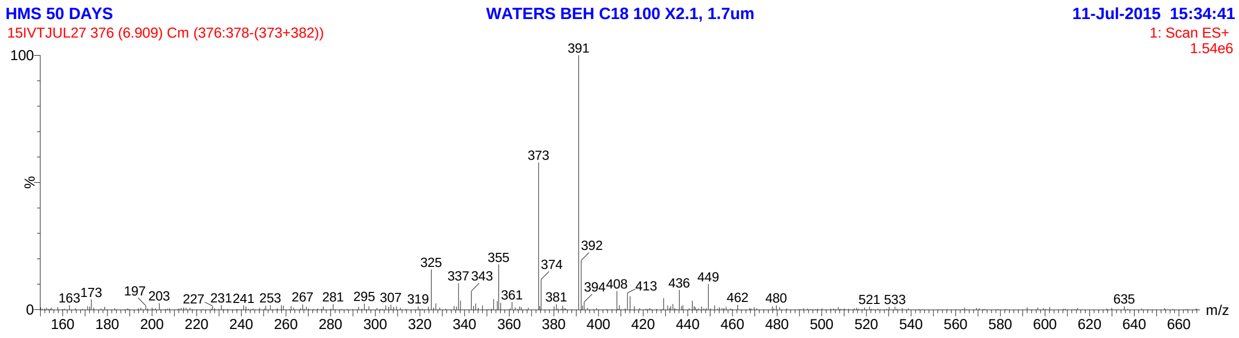

LC-ESI-MS Spectrum of coroglaucigenin *m/z* 391 $[M+H]^+$

m/z	Annotation	Mass difference	Confirmation remark
391	$[M+ H]^+$	1Da	Molecular weight 390
408	$[M+ NH_4]^+$	18 Da	Da

Accurate mass observed	Annotation	Calc. mass of coroglaucigenin $[M+ H]^+$	Mass difference In mmu	Confirmation remark
391.2478	$[M+ H]^+$	391.2479	0.0001	Molecular formula of the compounds $C_{23}H_{35}O_5^+$

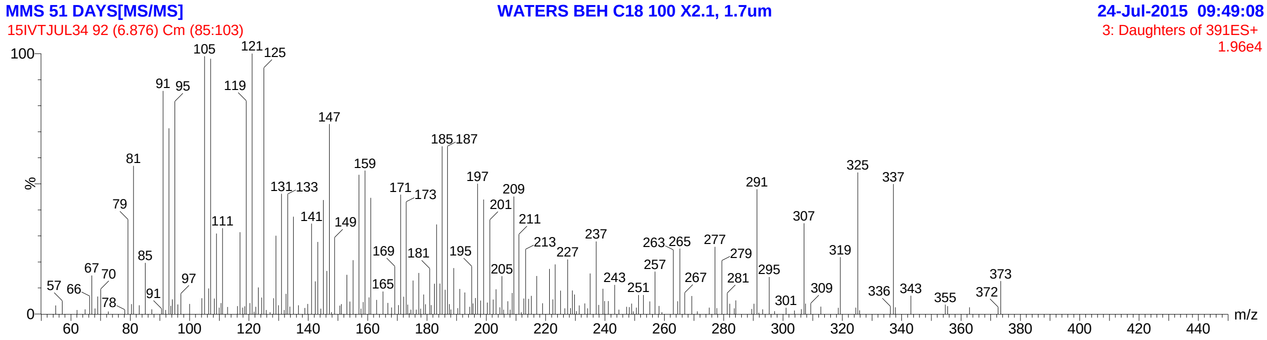

LC-ESI-MS/MS Spectrum of coroglaucigenin *m/z* 391 [M+H]$^+$ recorded at 30-20ev collision energy [characteristic fragment ions from genin observed *m/z* 373, 355, 337, 325]

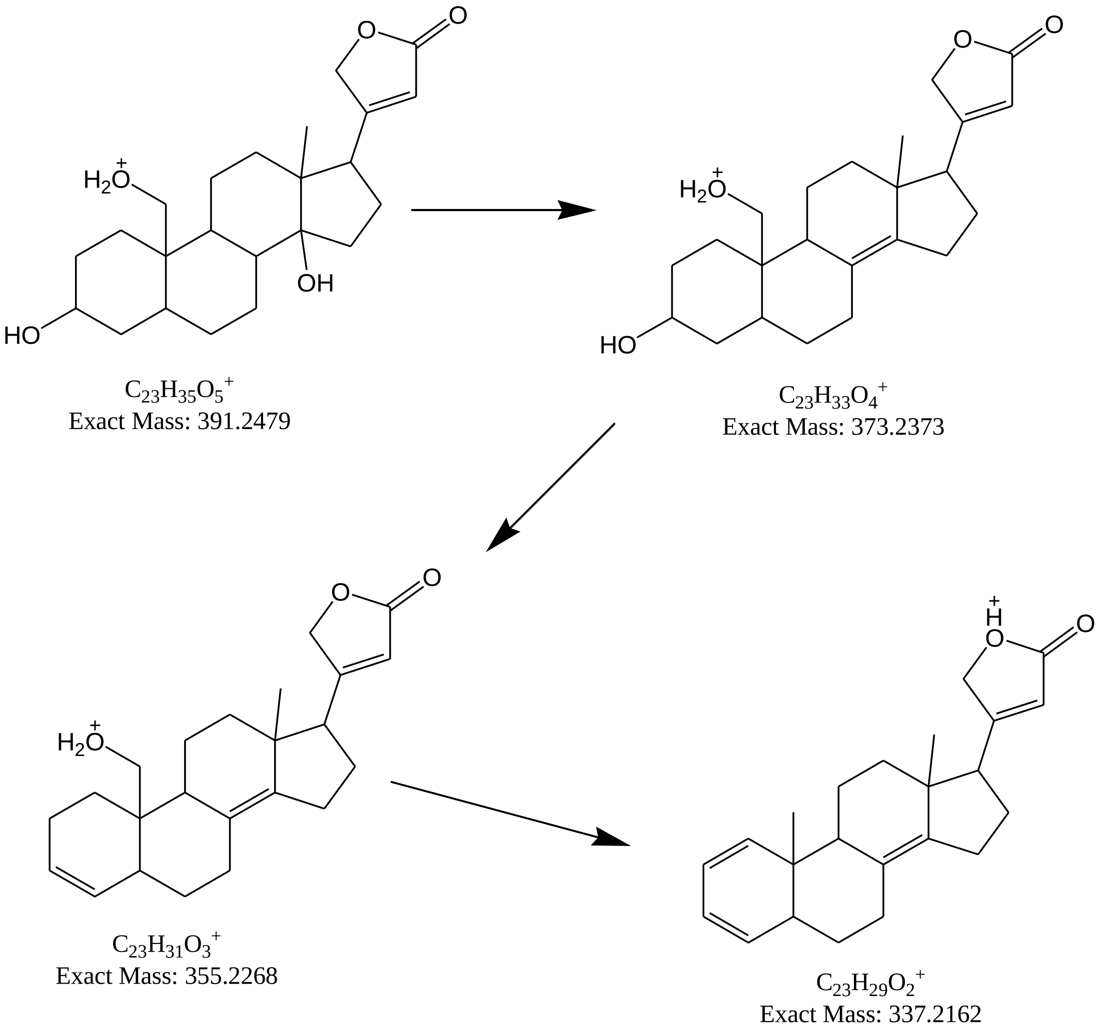

Proposed fragmentation pattern coroglaucigenin *m/z* 391 [M+H]$^+$

S. No.	RT	M.W.	Identified	*m/z* (Characteristic fragment ions of genin unit)	Genin unit	Sugar unit
04	7.85	532	Calotropin	387,369,359,351,341,333,323	A	146

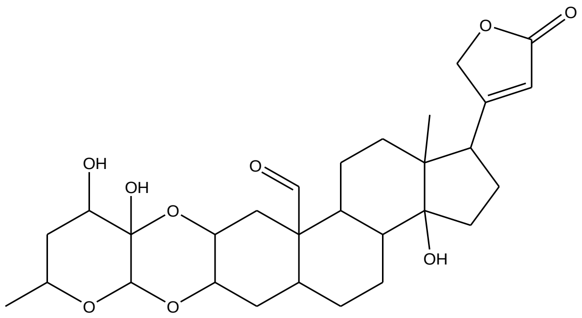

Calotropin
Chemical Formula: $C_{29}H_{40}O_9$
Exact Mass: 532.2672
Molecular Weight: 532.6225

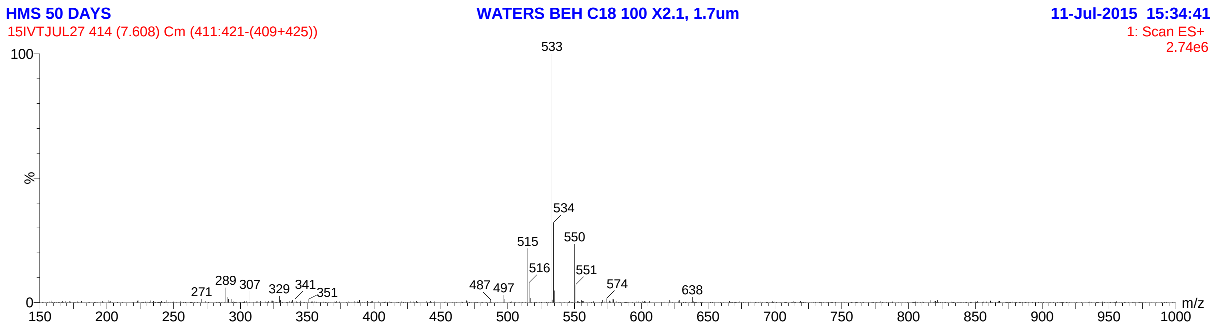

LC-ESI-MS Spectrum of calotropin *m/z* 533 $[M+H]^+$

m/z	Annotation	Mass difference	Confirmation remark
533	$[M+ H]^+$	1Da	Molecular weight 532
550	$[M+ NH_4]^+$	18 Da	Da

Accurate mass observed	Annotation	Calc. mass of Calotropin $[M+ H]^+$	Mass difference In mmu	Confirmation remark
533.2745	$[M+ H]^+$	533.2745	0.0000	Molecular formula of the compounds $C_{29}H_{41}O_9^+$

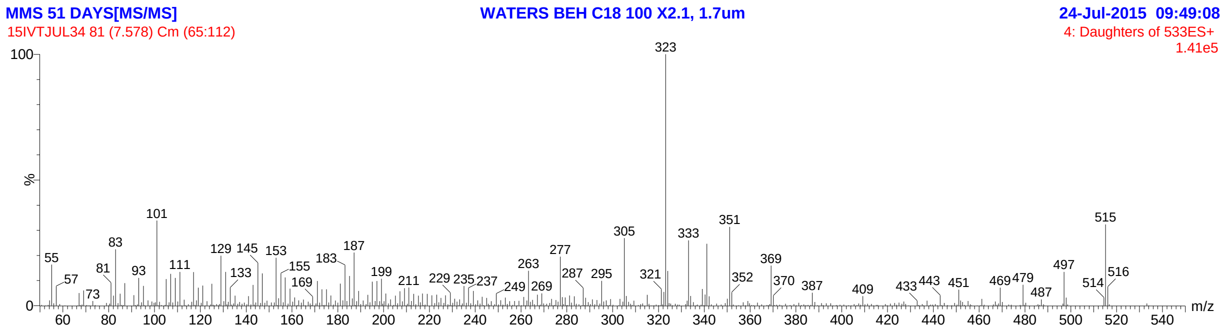

LC-ESI-MS/MS Spectrum of calotropin *m/z* 533 $[M+H]^+$ recorded at 30-20ev collision energy [characteristic fragment ions from genin observed *m/z* 387,369, 351,341,333,323,305]

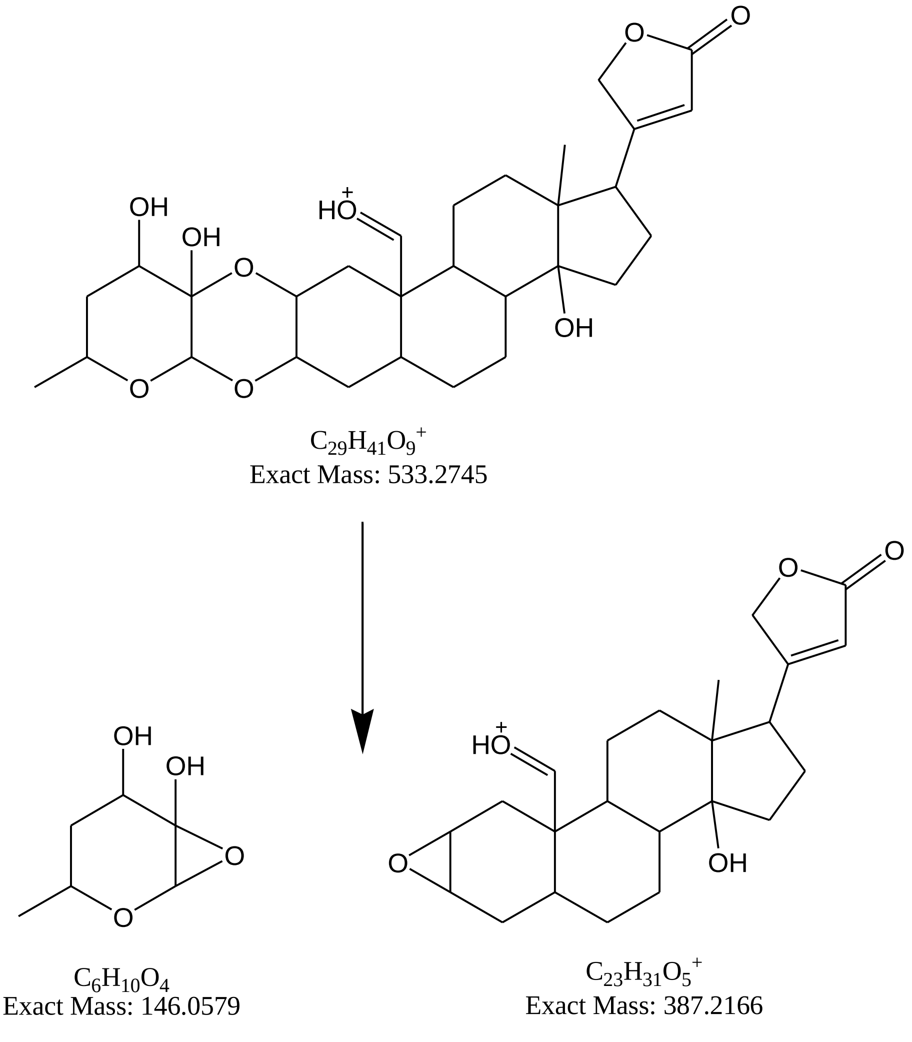

Proposed fragmentation pattern of calotropin m/z 533 $[M+H]^+$

S. No.	RT	M.W.	Identified	*m/z* (Characteristic fragment ions of genin unit)	Genin unit	Sugar unit
05	9.04	374	Uzarigenin	357,339,321,293	B	-

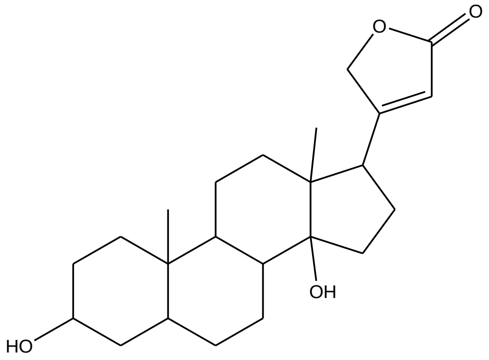

Uzarigenin
Chemical Formula: $C_{23}H_{34}O_4$
Exact Mass: 374.2457
Molecular Weight: 374.5137

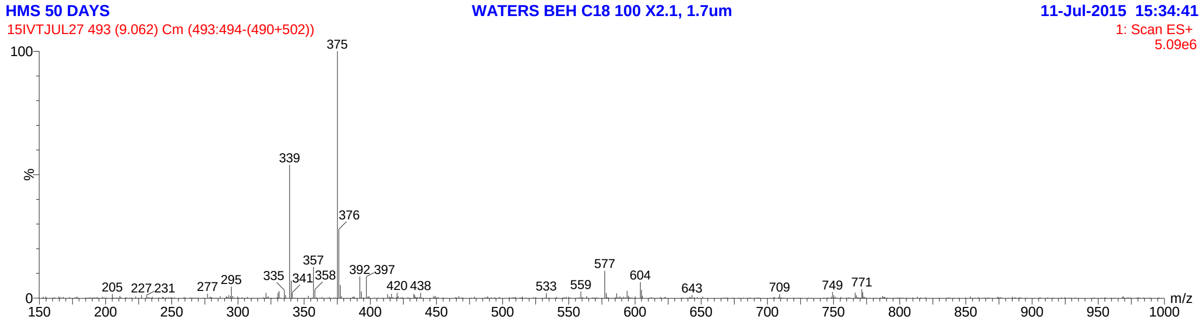

LC-ESI-MS Spectrum of uzarigenin *m/z* 375 $[M+H]^+$

m/z	Annotation	Mass difference	Confirmation remark
375	$[M+H]^+$	1Da	Molecular weight 374 Da
392	$[M+NH_4]^+$	18 Da	
397	$[M+Na]^+$	23 Da	

Accurate mass observed	Annotation	Calc. mass of uzarigenin $[M+H]^+$	Mass difference In mmu	Confirmation remark
375.2531	$[M+H]^+$	375.2530	0.0001	Molecular formula of the compounds $C_{23}H_{35}O_4^+$

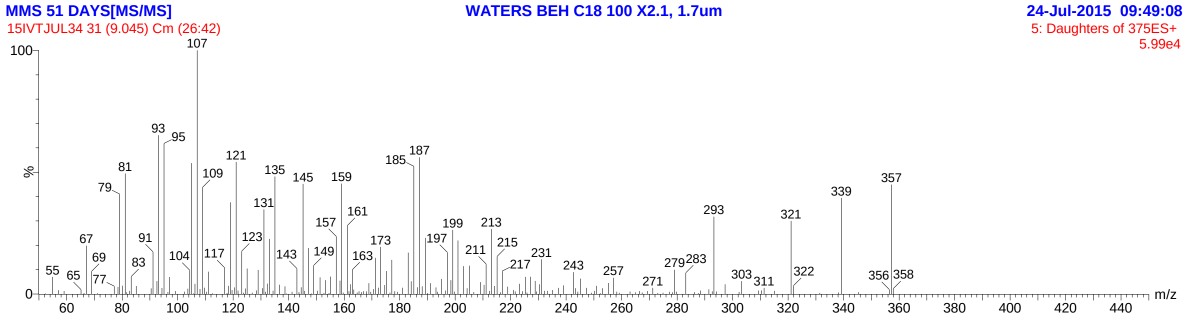

LC-ESI-MS/MS Spectrum of uzarigenin m/z 375 $[M+H]^+$ recorded at 30-20ev collision energy [characteristic fragment ions from genin observed *m/z* 357,339,321,293]

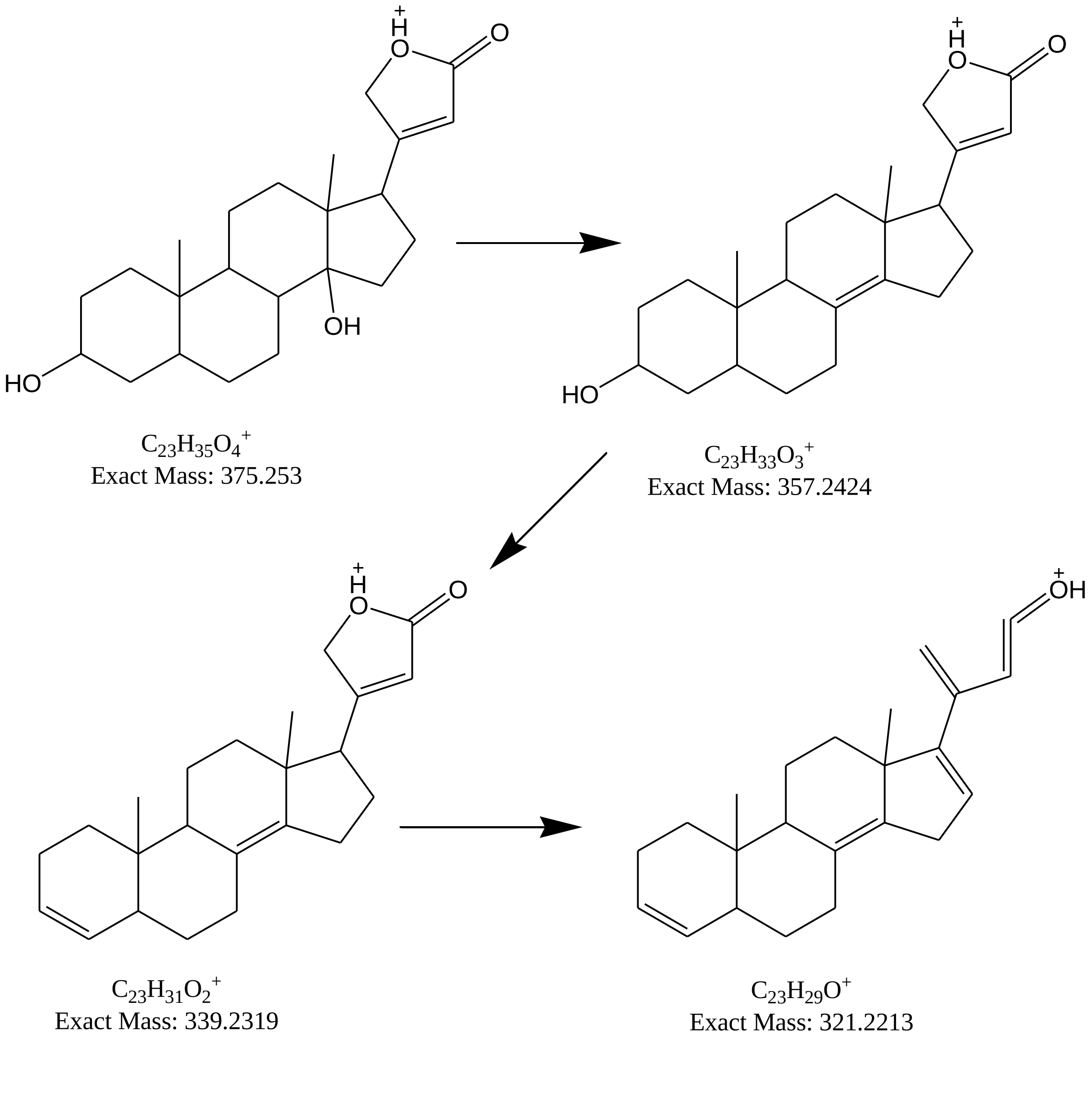

Proposed fragmentation pattern of uzarigenin m/z 375 $[M+H]^+$

S. No.	RT	M.W.	Identified	*m/z* (Characteristic fragment ions of genin unit)	Genin unit	Sugar unit
06	9.46	574	Asclepin	387,369,351,341,333,323	A	188

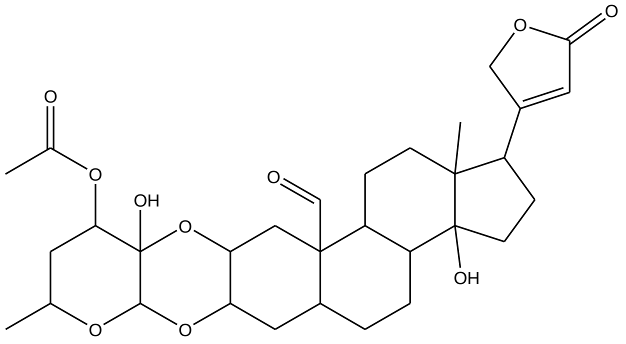

Asclepin
Chemical Formula: $C_{31}H_{42}O_{10}$
Exact Mass: 574.2778
Molecular Weight: 574.6592

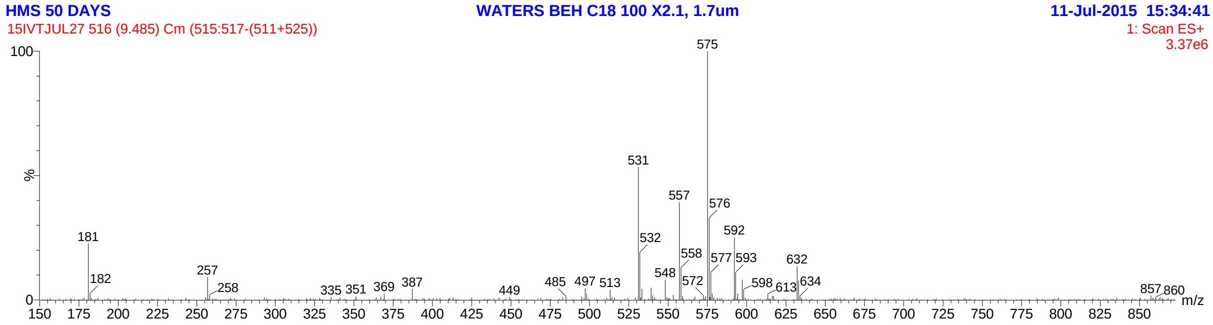

LC-ESI-MS Spectrum of asclepin m/z 575 $[M+H]^+$

m/z	Annotation	Mass difference	Confirmation remark
575	$[M+ H]^+$	1Da	Molecular weight 574 Da
592	$[M+ NH_4]^+$	18 Da	
597	$[M+ Na]^+$	23 Da	

Accurate mass observed	Annotation	Calc. mass of asclepin $[M+ H]^+$	Mass difference In mmu	Confirmation remark
575.2850	$[M+ H]^+$	575.2051	0.0001	Molecular formula of the compounds $C_{31}H_{43}O_{10}^+$

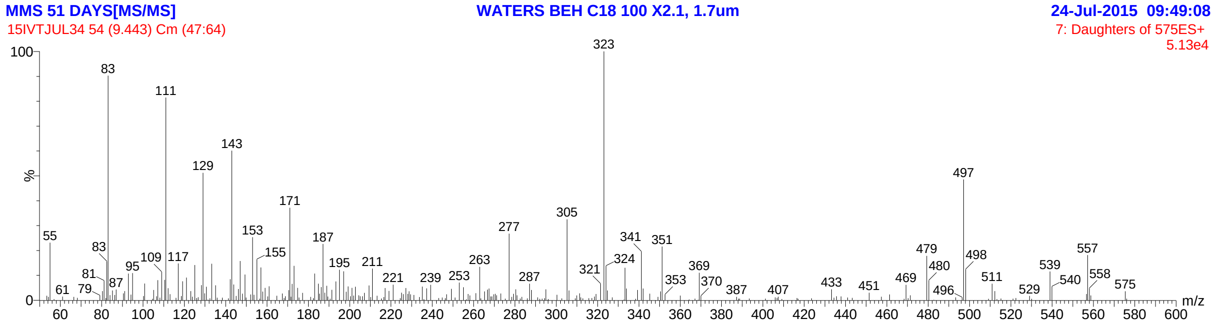

LC-ESI-MS/MS Spectrum of asclepin m/z 575 $[M+H]^+$ recorded at 30-20ev collision energy [characteristic fragment ions from genin observed m/z 387,369,351,341,333,323,305]

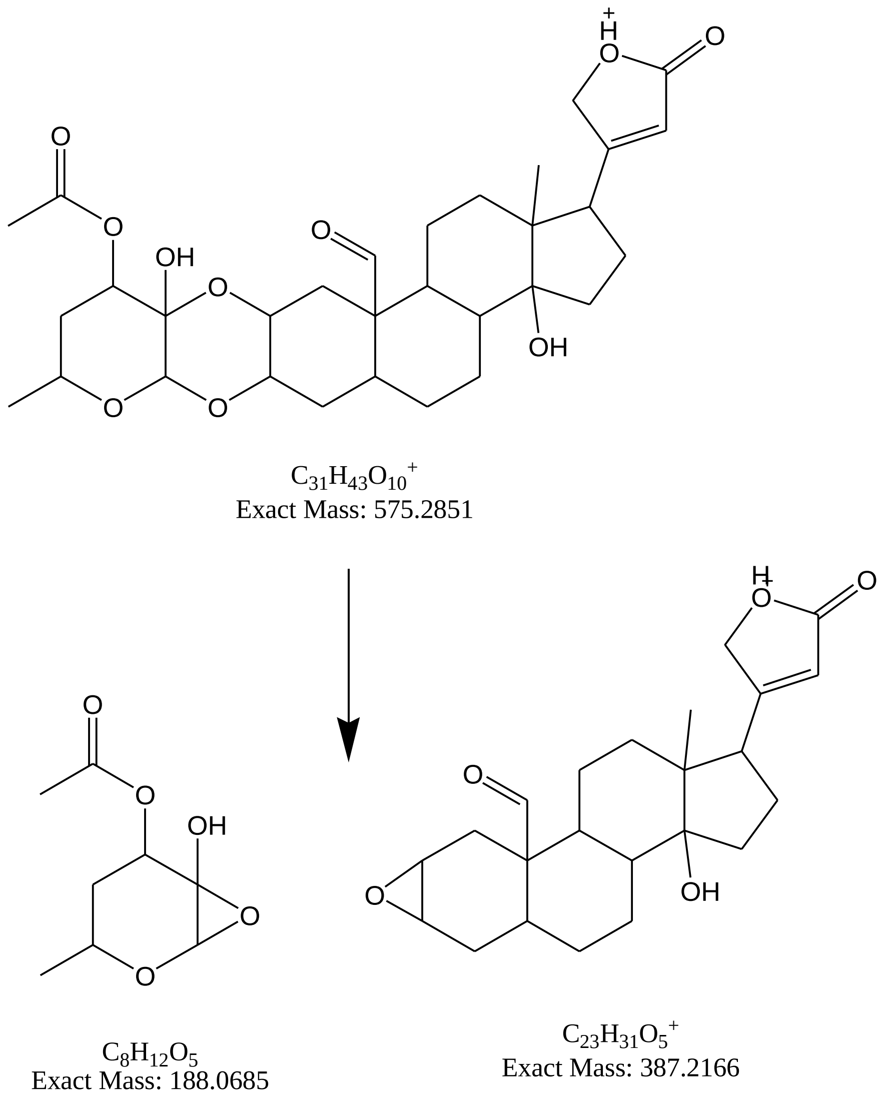

Proposed fragmentation pattern of asclepin m/z 575 $[M+H]^+$

S. No.	RT	M.W.	Identified	*m/z* (Characteristic fragment ions of genin unit)	Genin unit	Sugar unit
07	9.47	530	Uscharidin	369,351,341,333,323,305	A	144

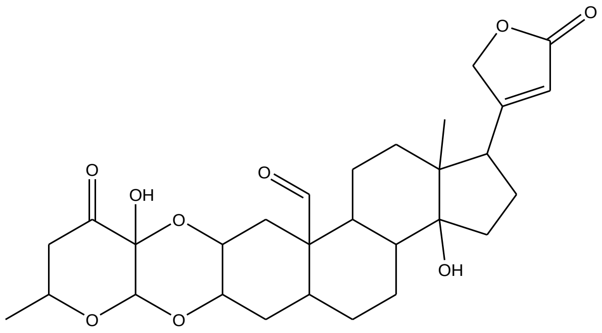

Uscharidin
Chemical Formula: $C_{29}H_{38}O_9$
Exact Mass: 530.2516
Molecular Weight: 530.6066

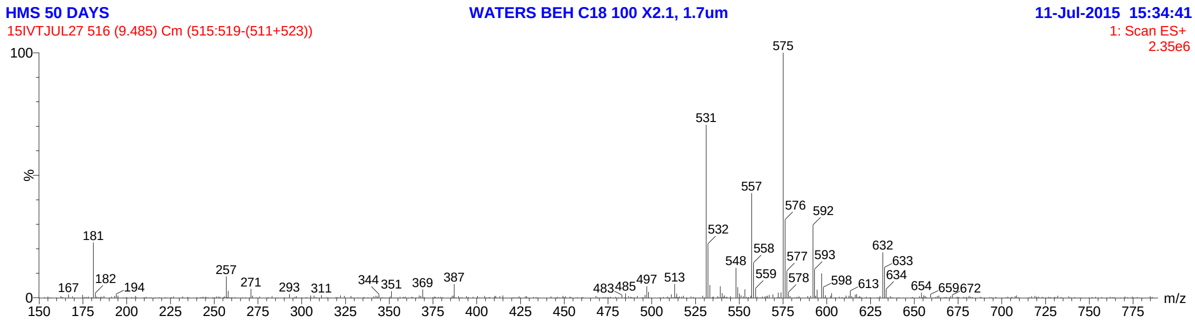

LC-ESI-MS Spectrum of uscharidin m/z 531 $[M+H]^+$

m/z	Annotation	Mass difference	Confirmation remark
531	$[M+H]^+$	1Da	Molecular weight 530 Da
548	$[M+NH_4]^+$	18 Da	
553	$[M+Na]^+$	23 Da	

Accurate mass observed	Annotation	Calc. mass of uscharidin $[M+H]^+$	Mass difference In mmu	Confirmation remark
531.2589	$[M+H]^+$	531.2589	0.0000	Molecular formula of the compounds $C_{29}H_{39}O_9^+$

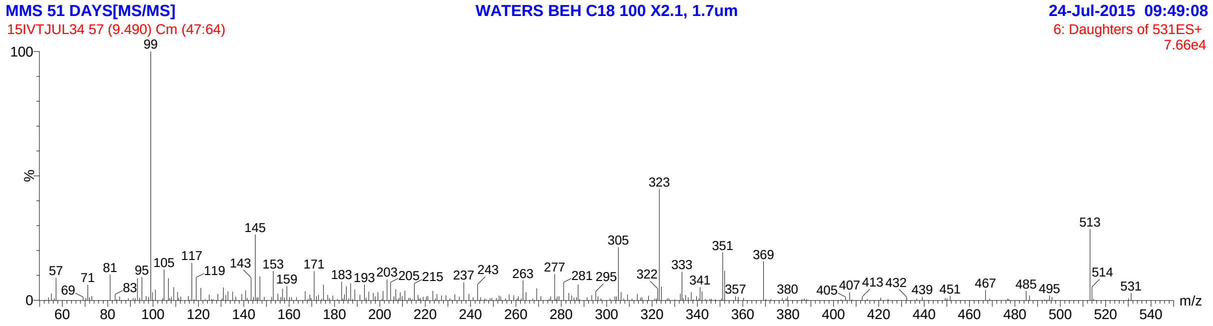

LC-ESI-MS/MS Spectrum of uscharidin m/z 531 $[M+H]^+$ recorded at 30-20ev collision energy [characteristic fragment ions from genin observed m/z 369,351,341,333,323,305]

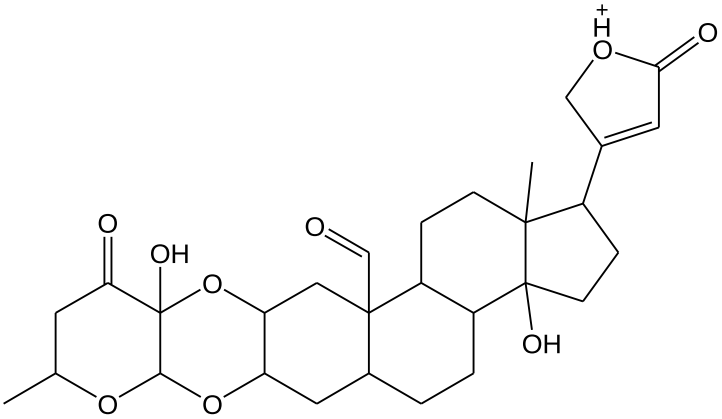

$C_{29}H_{39}O_9^+$
Exact Mass: 531.2589

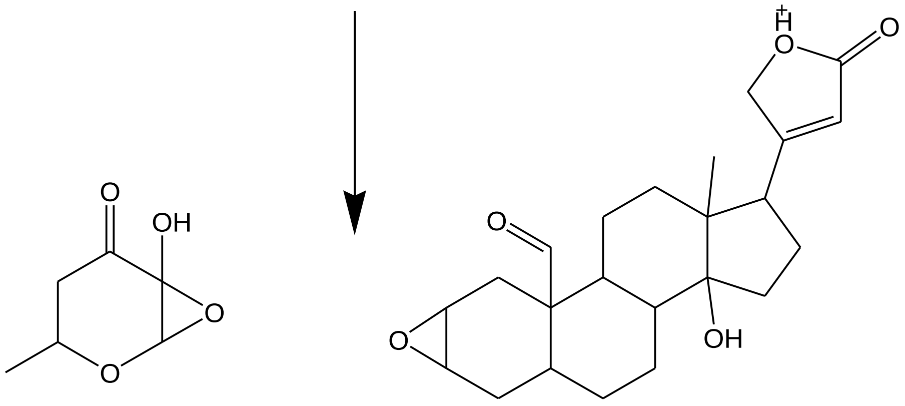

$C_6H_8O_4$
Exact Mass: 144.0423

$C_{23}H_{31}O_5^+$
Exact Mass: 387.2166

Proposed fragmentation pattern of uscharidin m/z 531 $[M+H]^+$

S. No.	RT	M.W.	Identified	*m/z* (Characteristic fragment ions of genin unit)	Genin unit	Sugar unit
08	9.90	586	Uscharin	369,351,341,333,323,305	A	201

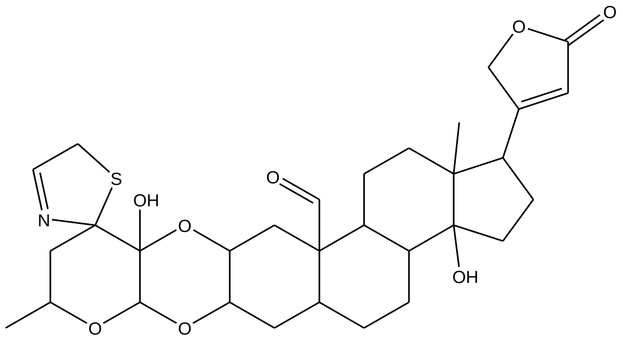

Uscharin
Chemical Formula: $C_{31}H_{41}NO_8S$
Exact Mass: 587.2553
Molecular Weight: 587.7241

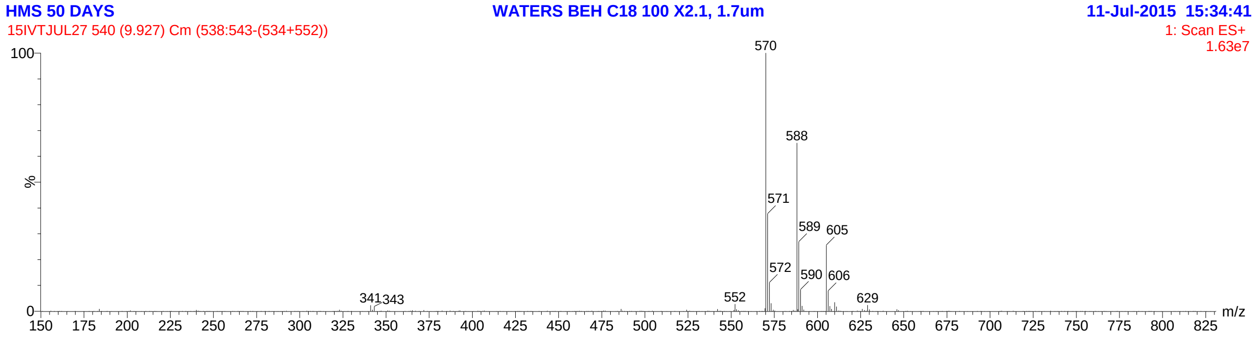

LC-ESI-MS Spectrum of uscharin m/z 588 $[M+H]^+$

m/z	Annotation	Mass difference	Confirmation remark
588	$[M+ H]^+$	1Da	Molecular weight 587
605	$[M+ NH_4]^+$	18 Da	Da

Accurate mass observed	Annotation	Calc. mass of uscharin $[M+ H]^+$	Mass difference In mmu	Confirmation remark
588.2629	$[M+ H]^+$	588.2626	0.0003	Molecular formula of the compounds $C_{31}H_{42}NO_8S^+$

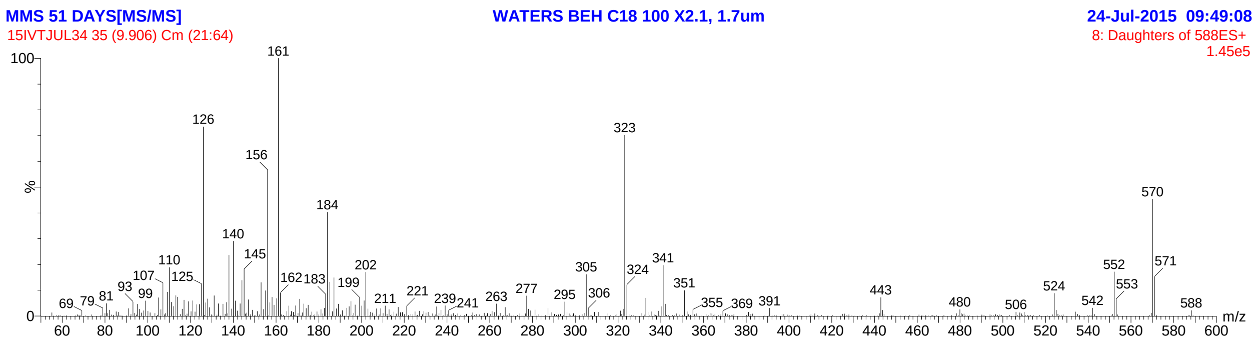

LC-ESI-MS/MS Spectrum of uscharin m/z 588 $[M+H]^+$ recorded at 30-20ev collision energy [characteristic fragment ions from genin observed m/z 369,351,341,333,323,305]

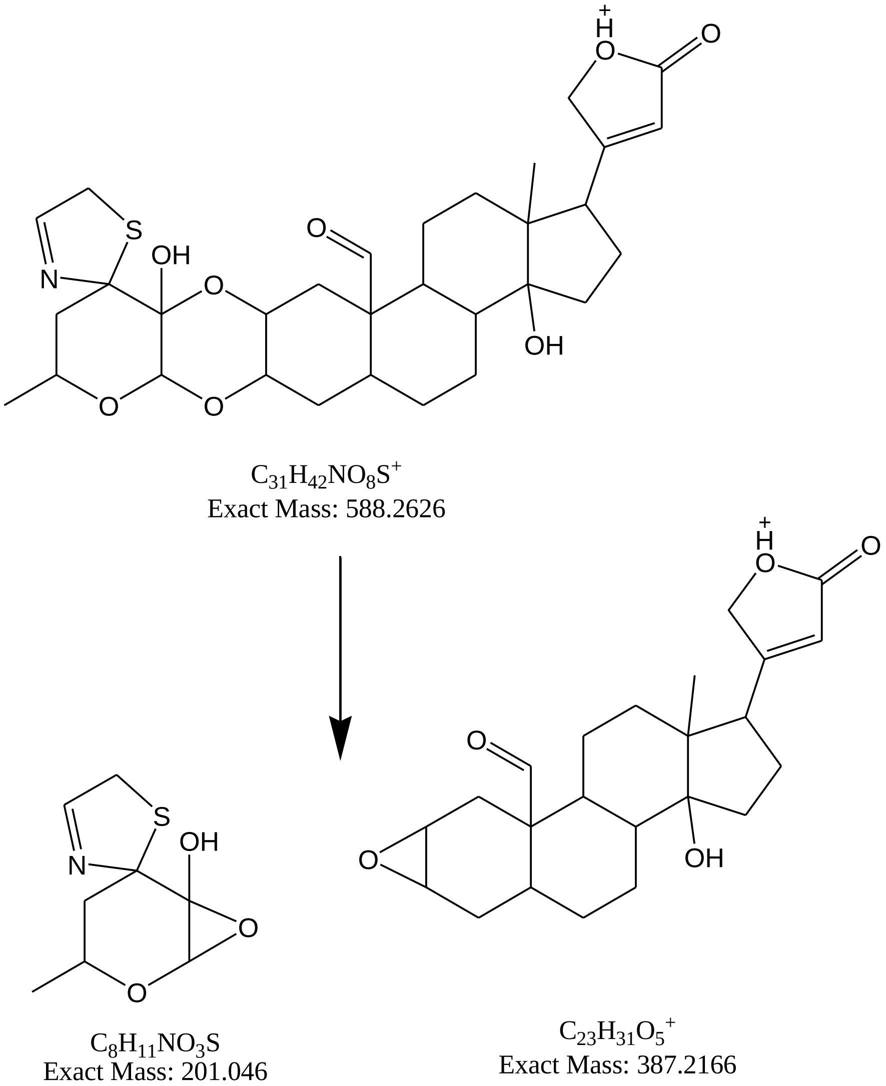

Proposed fragmentation pattern of uscharin m/z 588 [M+H]

Supplementary Table 1. Relative peak area intensity of metabolites measured by UPLC/Q-TOF MS.

S.No	Metabolite	Area (Mean+-SD)						
		3 month	6 month	9 month	12 month	Leaf(3 month)	Stem(3 month)	Root(3 month)
1	Uzarigenin	146941±5673	110247±4309	117506±8178	137993±6232	13194±2540	192415±10980	8212±1342
2	Calotropagenin	40729±5601	15062±4442	33236±5463	12365±2343	24302±5608	57545±14327	15615±1450
3	Coroglaucigenin	18768±2567	17318±2678	3998±986	1564±543	5261±634	1282±324	256±99
4	Frugoside	233200±9989	55684±4532	82267±6533	10457±1053	26937±4538	389985±25609	62190±4390
5	Uscharidin	114256±5523	40964±6732	35717±6623	75487±2365	103472±7908	266356±11123	38905±16700
6	Uscharin	1810726+4536	333477±7743	592055±5633	1346303±3783	857564±85750	1500333±15009	952749±98780
7	Ascelpin	113935±2323	47581±4288	61529±6234	88052±7723	59291±5532	135970±43308	73342±8900
8	Calactin	297240±5678	88170±9823	128451±9982	257425±2673	121638±1800	376483±35876	172995±9990

Supplementary Table 2- Plausible Unigenes involved in cardiac glycosides biosynthesis.

S.No.	Name of Enzyme	EC no.	Unigene ID	No. of transcripts
**1**	DXS, 1-deoxy-D-xylulose-5-phosphate synthase	EC:2.2.1.7	16078,11240	2
**2**	DXR, 1-deoxy-D-xylulose-5-phosphate reductoisomerase	EC:1.1.1.267	2570	2
**3**	MCT, 2-C-methyl-D-erythritol 4-phosphate cytidylyltransferase (ISP)	EC:2.7.7.60	3403	1
**4**	CMK, 4-diphosphocytidyl-2-C-methyl-D-erythritolkinase(4diphosphocytidyl-2C-methyl-D-erythritol synthase)	EC:2.7.1.148	6895	1
**5**	MDS, 2-C-methyl-D-erythritol 2,4-cyclodiphosphate synthase	EC:4.6.1.12	18970	1
**6**	HDS, 4-hydroxy-3-methylbut-2-en-1-yl diphosphate synthase	EC:1.17.7.1	1922	2
**7**	HDR, 4-hydroxy-3-methylbut-2-enyl diphosphate reductase	EC:1.17.1.2	1156	1
**8**	AACT, acetyl-CoA C-acetyltransferase	EC:2.3.1.9	12190	2
**9**	HMGS, hydroxymethylglutaryl-CoA synthase	EC:2.3.3.10	1140	4
**10**	HMGR, hydroxymethylglutaryl-CoA reductase	EC:1.1.1.34	1054	4
**11**	MK, mevalonate kinase	EC:2.7.1.36	8210	3
**12**	PMK, phosphomevalonate kinase	EC:2.7.4.2	1929	2
**13**	MPD, diphosphomevalonate decarboxylase	EC:4.1.1.33	2203	5
**14**	IDI, isopentenyl-diphosphate delta-isomerase	EC:5.3.3.2	6514	7
**15**	GGPPS1, geranylgeranyl pyrophosphate synthase 1	EC:2.5.1.1/10	568	6
**16**	FDPS1, farnesyldiphosphate synthase 1	EC:2.5.1.1 2/10	568	6
**17**	SQS1, squalene synthase	EC:2.5.1.21	12515	4
**18**	SMO, squalene monooxygenase	EC 1.14.13.13 2	3321,811,18 064	12
**19**	CAS1, cycloartenol synthase 1	EC:5.4.99.8	2371,8367	10
**20**	SMT1, sterol 24-C-methyltransferase	EC:2.1.1.41	7133	2
**21**	CPI1, cyclopropyl isomerise;CPI1; cycloeucalenol cycloisomerase	EC:5.5.1.9	807	7
**22**	CYP51G1, cytochrome P450, family 51,subfamily A (sterol 14-demethylase)	EC:1.14.13.70	8978	4
**23**	FK, delta14-sterol reductase	EC:1.3.1.70	8353	7
**24**	HYD1, cholestenol delta-isomerase	EC:5.3.3.5	3051	1
**25**	SMT2, sterol methyltransferase 2	EC:2.1.1.143	2514	1
**26**	STE1, C-5 sterol desaturase	EC:1.14.2.6	172	4
**27**	DWF5, sterol delta7 reductase	EC:1.3.1.21	1261	4
**28**	DWF1, delta24-sterol reductase,(delta14-sterol reductase)	EC:1.3.1.72	2522	1
**29**	3βHSD, 3-beta-hydroxysteroid-dehydrogenase	-	5463, 14271, 845,303	15
**30**	5βPOR, progesterone 5β-reductase	-	1572	1
**31**	GH, glucohydrolase	-	9455,12988	4

**32**	GT, glycosyltransferase/glucuronosyltransferase<br>UGT,UDPglycosyltransferase/glucuronosyltransferase	-	9455,12988,16256,889,794,2312,6827,15669,1971,6604,9044,14107,16749,6297,14001,3348,11001,13521,16372,2170,8004,5351,9397,1021,19905,3824,4441,9684,4803,8678,22663,13316,7329,4933,5124,3223,8578,9784,4417,8515,6875,10638,173,3747,5219,517,7752,10217,10597,5130,2166,15366,5087,2314,4704,4060,5333,7748,11734,1133,4951,7455,16503,6830	125
**33**	Monooxygenases	-	248,426,429,591,619,811,1466,1647,1784,2419,2992,4204,5880,6582,7188,13087,14352,14542,16719,11371,8206,7319,7565,7582,2206,8421,8498,8914,9235,10089,10751,11372,12547,12715,12768,12818,17161,18175,13666,2994.	90

Supplementary table 3- Primer sequences used in qRT-PCR analysis

Name	Forward Primer	Reverse Primer
**ACTIN**	GCGTCGGAGGTGAGAGAG	AGAGTGTACGGGCCATGC
**DXS**	TGCAGCTGCCATAGACGATA	TCCTATCCCATTTCCACGAG
**DXR**	CCCTGATGCTGTCACTGTTG	CGCTGCTACTGTTGGCTTTA
**MCT**	TCGTTTATCCAGAAGGCAGTC	TTCACAACAGCACCGGAAG
**CMK**	TGATCTAGAACATCCTGCTTTTGA	ACACGCTGCTTCAACCTTTT
**MDS**	TCGTTTATCCAGAAGGCAGTC	TTCACAACAGCACCGGAAG
**HDS**	ACAATCAGAGTGTCTCTTACTGAACC	TGCTAACCTTCTACAGGGGTCT
**HDR**	CAGACTCGTCGGTGGTAGC	CGGAAACCTGTGTCTGTACG
**AACT**	CAGTGCGAATTTAGGCCAAG	TTAGGAATACCAGCGCCAAG
**HMGS**	TTTCCTCCTACTTGCATCCAG	TTCCCTTTGCTTGCTCCA
**HMGR**	CCATTTCGCCCTCTCCTAA	CTTCTGAAGAATGCGAGGTGA
**MK**	ACAAGTTGTCTTCCAAGTTGACTG	TGGCAATAGTGTCAACACACAG
**PMK**	CCCGTCAATGGTAGGAGCTA	TTCTAGAGAATTTTGGGGGTCA
**MPD**	TTCCCATCTCTGTACCACCAC	CCACATACGATCCTGCGTAA
**IDI**	ACCGGCGGTTACACCTATC	GCAGCAACAGAAGAAAAGCA
**SQS**	TAAGGTTTCGAGGAGCTTTGC	GCGTCCCGAAGATCAGTATC
**SMO**	AACATGCGCCATCCTTTAAC	GATCACGAAGGACAACAATGTCT
**SQE**	CTTTTCTTGTTTCTTTATCTGGGTTT	TGGTTCAAATTTAGAAGAAAGCTTAGA
**CAS 1**	ACTGGCGGAGATCGAAAAG	CGCTGTGCTTTTTCTCGAAT
**SMT1**	CCTCCGGGAAAGCATTAAG	CCAGGTCTCAGGCCTAGTTG
**CPI1**	CACAGTTTTGGGTGCTTCCT	TGAGTGAGGAGAAAAGTAGTATGTGG
**CYP**	GCCGGAGCATTCTCATACAT	TGGCTCTCCAAGACATCCAT
**HYD1**	GAAAATCTCAAAGTTGGATAGGATTC	CATGTGGGTTAGGCCAGTG
**SMT2**	AAATGGCCGTGGATCTATTG	CGCATCCAACATCGAGAAT
**STE 1**	TGCAAATGTCTGTTGCAATG	TCACGTATTCAGAGACTGTAGGAAG
**DWF 5**	GAAAAAGGGTTGAGGGTCCTA	TGCTTGCAAACCATTAGCC
**DWF1**	GACAAGGAGAGGATGGTTGC	CCGGGATATCTGACCCATATT
**POR**	ATGAATCTAGTTGGTACCATTTGTGT	GGAAACTTCAATGGCACTCC
**LS**	TTTGATCCTAATGCCGGAAC	ATTCCCGGCGAACTCTTT
**GH**	TTGGTGACGAGAACGAGGTT	ACCAAATTCCCATTGTACGC
**GT**	CCCTGTAAATTGCATGCTGA	GGAGTGTTAAGGAAAGTGACGTG
**MO**	TCCCACCAAGAAACTTCCAC	TGTGAATGTGTCCGATGATTG

Supplementary Table no 4- Expanded names of genes for figures 6 and 7.

**Abbreviation**	**Full name**
**DXS**	1-Deoxy-D-xylulose-5-phosphate synthase
**DXR**	1-Deoxy-D-xylulose-5-phosphate reductoisomerase
**MCT**	2-C-methyl-D-erythritol4-phosphate cytidylyltransferase
**CMK**	4-Diphosphocytidyl-2-C-methyl-D-erythritolkinase;
**MDS**	2-C-methyl-D-erythritol 2,4-cyclodiphosphate synthase
**HDS**	4-Hydroxy-3-methylbut-2-en-1-yldiphosphate synthase
**HDR**	4-Hydroxy-3-methylbut-2-enyl diphosphate reductase
**AACT**	AcetylCoAacetyltransferase
**HMGS**	HydroxymethylglutarylCoAsynthase
**HMGR**	Hydroxymethylglutaryl-CoA reductase
**MK**	Mevalonate kinase
**PMK**	Phosphomevalonate kinase
**MPD**	Diphosphomevalonate decarboxylase
**IDI**	Isopentenyl-diphosphate delta-isomerase
**GGPPS1**	Geranylgeranyl pyrophosphate synthase 1
**FDPS1**	Farnesyldiphosphate synthase 1
**SQS**	Squalene synthase
**SMO**	Squalene monooxygenase
**CAS**	Cycloartenol synthase
**SMT1**	Sterol 24-C-methyltransferase
**CPI1**	Cyclopropyl isomerase
**CYP51G1**	Cytochrome P450, family 51,subfamily A(sterol14demethylase)
**FK**	Delta14-sterol reductase
**HYD**	Cholestenol delta-isomerase
**SMT2**	Sterol methyltransferase 2
**STE**	C-5 sterol desaturase
**DWF5**	Sterol delta7 reductase
**DWF1**	Delta24-sterolreductase
**3βHSD**	3beta-hydroxy-steroid-dehydrogenase
**POR**	Progesterone reductase
**OR**	Oxidoreductase
**MaT**	Malonyltransferase
**HOX**	Hydroxylase
**GH**	Glucohydrolase
**MO**	Monooxygenases
**GT**	Glycosyltransferase/glucuronosyltransferase
**UGT**	UDP-glycosyltransferase/ glucuronosyltransferase
